# CT-based radiomics in predicting the efficacy of preoperative neoadjuvant chemoimmunotherapy for non-small cell lung cancer: a systematic review and meta-analysis

**DOI:** 10.3389/fimmu.2026.1753166

**Published:** 2026-02-10

**Authors:** Hongyang Chen, Bingjie Fan, Mengqi Yuan, Dandan Wang, Chenxi Qiao, Na Qiu, Xiaomin Quan, Wei Hou

**Affiliations:** 1 Department of Graduate School, Beijing University of Chinese Medicine, Beijing, China; 2 Department of Oncology, Guang'anmen Hospital, China Academy of Chinese Medical Sciences, Beijing, China; 3 Capital Medical University, Beijing, China; 4 Beijing Hospital of Traditional Chinese Medicine, Beijing, China; 5 China Academy of Chinese Medical Sciences, Beijing, China; 6 Faculty of Chinese Medicine and State Key Laboratory of Mechanism and Quality of Chinese Medicine, Macao University of Science and Technology, Macao, Macao SAR, China

**Keywords:** computed tomography, meta-analysis, neoadjuvant chemoimmunotherapy, non-small cell lung cancer, prediction model

## Abstract

**Introduction:**

Neoadjuvant chemoimmunotherapy significantly improves surgical resection rates, major pathological response rates (MPR), pathological complete response rates (pCR), and survival rates in patients with resectable NSCLC. Through systematic reviews and meta-analyses, we examined the diagnostic value of CT-based predictive models in predicting neoadjuvant chemoimmunotherapy treatment outcomes for NSCLC.

**Method:**

PubMed, Embase, Web of Science databases, China National Knowledge Infrastructure, and Wanfang were systematically searched up to January 12, 2026. To assess study risk of bias and quality, we employed the Quality Assessment of Diagnostic Accuracy Studies (QUADAS) tool and the Radiomics Quality Score version 2.0(RQS). Diagnostic accuracy of radiomics for detecting neoadjuvant chemoimmunotherapy pathological response in NSCLC patients was evaluated by calculating the area under the curve (AUC), sensitivity, specificity, and accuracy for each study.

**Results:**

The meta-analysis analyzed 17 studies with 4,510 individual subjects. The pooled AUC, sensitivity, and specificity of internal validation models were 0.81, 0.79, and 0.69, respectively. The pooled AUC, sensitivity, and specificity of external validation models were 0.80, 0.75, and 0.73, accordingly. Subgroup analyses revealed that models using deep learning (DL) algorithms demonstrated superior sensitivity (internal: 0.79, 95% CI: 0.73-0.85; external: 0.77, 95% CI: 0.72-0.82) and specificity (internal: 0.79, 95% CI: 0.74-0.85; external: 0.73, 95% CI: 0.68-0.78) compared to those using machine learning (ML). Models predicting MPR exhibited higher sensitivity in internal validation (0.82, 95% CI: 0.77-0.86), while showing higher specificity in external validation (0.76, 95% CI: 0.72-0.81). In contrast, models predicting pCR demonstrated the opposite pattern. Features selected using the intraclass correlation coefficient (ICC) demonstrated significantly higher pooled sensitivity (internal: 0.85, 95% CI: 0.80-0.89; external: 0.81, 95% CI: 0.76-0.87) and specificity (internal: 0.70, 95% CI: 0.63-0.78; external: 0.77, 95% CI: 0.71-0.82) compared to non-ICC-selected features. When stratified by the median Radiomics Quality Score (RQS ≥ 41.07%), higher-scoring studies were associated with lower pooled sensitivity (internal: 0.78, 95% CI: 0.73-0.84; external: 0.71, 95% CI: 0.66-0.76) but a trend toward higher specificity. Finally, models based on two-dimensional regions of interest (2D ROI) demonstrated higher pooled sensitivity (internal: 0.86, 95% CI: 0.80-0.92; external: 0.87, 95% CI: 0.79-0.96) and specificity in external validation (0.80, 95% CI: 0.68-0.91).

**Conclusion:**

Due to its good diagnostic accuracy, widespread use, and low cost, CT-based radiomics can be used to predict the efficacy of neoadjuvant chemoimmunotherapy in NSCLC preoperatively.

**Systematic Review Registration:**

https://www.crd.york.ac.uk/prospero/, identifier (CRD420251174128).

## 1 Introduction

Lung cancer is one of the most commonly diagnosed cancers and the leading cause of cancer-related deaths globally. Non-small cell lung cancer (NSCLC) accounts for approximately 85% of all lung cancer cases. Surgery is the standard treatment for resectable NSCLC. Unfortunately, even after radical resection, early-stage disease still carries a high risk of recurrence and mortality (1). A meta-analysis demonstrated that neoadjuvant chemotherapy significantly improved overall survival(OS), time to distant recurrence, and recurrence-free survival(RFS) in patients with resectable NSCLC (2). However, compared to surgery alone, neoadjuvant chemotherapy yields only a 5% to 6% absolute difference in 5-year RFS and OS rates—a figure that remains unsatisfactory. In the context of early-stage or locally advanced NSCLC, neoadjuvant chemoimmunotherapy has emerged as a significant research direction in solid tumor treatment. Neoadjuvant therapy combining PD-1/PD-L1 blockade with chemotherapy has demonstrated significant improvements in resection rate, major pathological response (MPR) rate, pathological complete response (pCR) rate, and survival rates among patients with resectable NSCLC (3, 4). A meta-analysis demonstrated that patients receiving neoadjuvant or perioperative chemotherapy combined with immunotherapy exhibited significantly superior event-free survival (EFS) compared to those receiving neoadjuvant chemotherapy alone [hazard ratio (HR) 0.58, 95% confidence interval (CI) 0:0.51-0.66] (5).

In most trials involving neoadjuvant chemoimmunotherapy for NSCLC, MPR serves as a surrogate endpoint for OS and disease-free survival (DFS). The MPR rates observed in current neoadjuvant chemoimmunotherapy clinical studies vary significantly, ranging from 18% to 83% (6). Clinical trials have established that MPR and pCR serve as key surrogate endpoints for evaluating chemoimmunotherapy efficacy. However, a substantial proportion of patients fail to benefit from neoadjuvant chemoimmunotherapy. The occurrence of immune-related adverse events and relatively low clinical response rates in patients with NSCLC underscore the limitations of neoadjuvant chemoimmunotherapy (7, 8). High intratumoral heterogeneity may be a primary driver of this therapeutic disparity (9, 10). The peritumoral microenvironment, characterized by abundant tumor-infiltrating lymphocytes and tumor-associated macrophages, can modulate responses to immunotherapy (11, 12). Current clinical approaches to obtaining information on intratumoral heterogeneity and the peritumoral microenvironment involve invasive tissue biopsies, limiting the feasibility of repeated needle biopsies during neoadjuvant chemoimmunotherapy. Consequently, there is an urgent need for an alternative method to predict response to neoadjuvant treatment. Accurate prediction of neoadjuvant chemoimmunotherapy outcomes enables timely formulation of appropriate treatment strategies, which avoids potential undertreatment or overtreatment and prevents unnecessary complications associated with surgery. Programmed Death-Ligand 1/Programmed Death-1 (PD-L1/PD-1) represents the most critical immunotherapy target in NSCLC. However, recent studies demonstrate that PD-L1 expression exhibits significant dynamic changes throughout disease progression. This may result in initial test results failing to accurately reflect the immune status at the time of treatment, thereby compromising predictive reliability. The lack of standardized strategies for PD-L1 testing—including inconsistent timing of assessment, number of biopsies, and specimen interpretation criteria—further limits its predictive value. In summary, reliable biomarkers for predicting pathological response after neoadjuvant therapy in resectable NSCLC are currently unavailable (13).

Computed tomography (CT), as a non-invasive imaging modality, has been widely adopted for preoperative evaluation of NSCLC and has demonstrated applicability in assessing pathological response to NSCLC treatment. However, numerous studies indicate that tumor size measurements based on CT scans cannot reliably predict pathological response following neoadjuvant therapy for resectable NSCLC (14). In the NADIM trial, 33% of patients with stable disease and 73% of those with radiographic partial response achieved pCR (15). This discrepancy is typically attributable to false positives. Lymphocytic infiltration can obscure lesions, as radiographic findings may fail to reflect actual tumor regression. To enhance the accuracy of predicting which patients will achieve postoperative MPR in NSCLC following neoadjuvant chemoimmunotherapy, the integration of multidimensional and high-throughput imaging features is essential.

With the advancement of artificial intelligence (AI), extracting radiomics or deep learning (DL) features from CT scans can provide additional information to enhance the diagnosis, treatment, and prognosis of lung cancer. A multicenter study predicted major pathological response to neoadjuvant chemotherapy and immunotherapy in non-small cell lung cancer using DL. After integrating clinical features into the DL score, the combined model demonstrated satisfactory performance in both internal validation (AUC: 0.77, 95% CI: 0.64-0.89) and external validation cohorts (AUC: 0.75, 95% CI: 0.62-0.87) (16). *Liu* et al. developed and validated a radiomics-based nomogram to predict major pathological response to neoadjuvant immunochemotherapy in potentially resectable NSCLC patients. The radiological-clinical combined model demonstrated excellent discriminatory performance, achieving AUCs of 0.84 (95% CI, 0.74-0.93) and 0.81 (95% CI, 0.63-0.98) (17). However, aggregated results derived from different predictive models, outcome measures, and model construction approaches appear to be inconsistent. Furthermore, the lack of standardized radiomics workflows limits the robustness and reproducibility of these models.

This study aims to systematically review and comprehensively summarize the application of CT in predicting the efficacy of preoperative neoadjuvant chemoimmunotherapy for NSCLC, focusing on its diagnostic performance, sensitivity, and specificity. It seeks to provide clinicians with a potential reference tool for evaluating the efficacy of neoadjuvant chemoimmunotherapy, thereby facilitating the development of personalized treatment strategies.

## 2 Materials and methods

### 2.1 Study protocol and registration

This systematic review and meta-analysis was conducted in accordance with the Preferred Reporting Items for Systematic Reviews and Meta-Analyses (PRISMA) guidelines (18), ensuring a structured and transparent methodology. The review protocol was registered and approved in the International Prospective Register of Systematic Reviews (PROSPERO) database (Registration ID: CRD420251174128).

### 2.2 Literature search strategy

In accordance with the PRISMA statement, two authors (HC and BF) independently performed a comprehensive database search using PubMed, Embase, Web of Science, China National Knowledge Infrastructure, and Wanfang as primary sources. The search covered the period from each database’s inception to January 12, 2026. A combination of Medical Subject Headings (MeSH) and keywords associated with CT, NSCLC, neoadjuvant therapy, chemoimmunotherapy, and prediction was employed. Additional target literature was identified by reviewing the references of included studies. The specific search strategy is detailed in **Supplementary Material**.

### 2.3 Inclusion criteria and screening

Inclusion and exclusion criteria were established based on the PICOS framework. The inclusion criteria were as follows: (1) Population (P): patients with NSCLC; (2) Intervention (I): an AI algorithm applied to CT imaging to predict neoadjuvant chemoimmunotherapy efficacy; (3) Comparator (C): patients not achieving pCR or MPR after neoadjuvant therapy; (4) Outcomes (O): efficacy (MPR/pCR status) presented in a 2×2 diagnostic contingency table; (5) Study design (S): machine learning (ML) or DL studies evaluating the diagnostic value of imaging for NSCLC, published in peer-reviewed journals.

The exclusion criteria were: (1) insufficient outcome data for analysis; (2) conference papers, case reports, systematic reviews, etc.; (3) unfinished studies or published research including unfinished data; (4) duplicate reports; (5) studies for which the full text was unavailable.

### 2.4 Study selection and data extraction

Two authors (HC and BF) recorded data in standardized spreadsheets. Any discrepancies were resolved through consultation with a third author (NQ). Extracted data included: (1) study characteristics: first author, publication year, country, data source, study design; (2) patient characteristics: sample size, training/validation set distribution, tumor stage; (3) radiomics-related parameters: imaging modality, tumor lesion segmentation method, region of interest (ROI) size, feature extraction software, feature type, and AI method; (4) validation methods; (5) model performance metrics: AUC values, sensitivity, specificity with 95% confidence intervals (95% CIs), and true positives (TP), false positives (FP), true negatives (TN), and false negatives (FN).

For studies that did not directly report sensitivity, specificity, or 2×2 tables, data were extracted from ROC curves using GetData Graph Digitizer 2.24 software (19). To mitigate selection bias, AUC values were derived from all validation set data within prediction models based on radiomics features, with stratified reporting for internal and external validation (20). Where two thresholds were used in model construction, the model built using the fixed threshold was selected.

### 2.5 Quality assessment

The Radiomics Quality Score version 2.0 (RQS) checklist and the QUADAS-2 tool were employed to evaluate the included studies (21, 22). Two authors (XQ and CQ) conducted independent assessments, resolving discrepancies through consultation with a third author (WH). The RQS 2.0 framework (accessed November 23, 2025, at https://www.radiomics.world/rqs2), proposed by *Lambin* and colleagues, was used to evaluate the quality of radiomics research reports across 42 assessment dimensions within 9 key domains (23, 24). The maximum achievable score is 56 points (100%). The QUADAS-2 tool was used to assess risk of bias and applicability in patient selection, index test, reference standard, and flow/timing. Responses were recorded as “yes,” “no,” or “unclear” in RevMan 5.4.

### 2.6 Data analysis

This meta-analysis employed STATA software (version 14) and Revman (version 5.4) for statistical analysis. A bivariate random-effects model was used to pool data across different validation datasets. Pooled sensitivity, specificity, positive likelihood ratio (PLR), negative likelihood ratio (NLR), and their corresponding 95% CIs were calculated. Summary receiver operating characteristic (SROC) curves and the area under the curve (AUC) were constructed to assess overall diagnostic performance (25). Diagnostic accuracy based on AUC values was categorized as: 0.90-1 (excellent), 0.80-0.90 (good), 0.70-0.80 (fair), 0.60-0.70 (poor), and 0.50-0.60 (very poor). Threshold effects were evaluated by calculating Spearman’s rank correlation coefficient between logit (sensitivity) and logit(1-specificity).

Heterogeneity was assessed using *I_2_* and Q statistics, with *I_2_* values categorized as low (0-50%), moderate (50-75%), or high (>75%). Forest plots displayed sensitivity and specificity across studies.

Meta-regression analyses and subgroup analyses of the radiomics model were performed to compare studies using different datasets, including clinical characteristics, model calibration methods, study design, data source, imaging modality, tumor lesion segmentation method, RQS score, ROI size, feature extraction software, model combination characteristics, AI method, and model validation methods.

To assess the influence of individual studies on the overall estimate, a sensitivity analysis was conducted by sequentially excluding one study at a time using a univariate diagnostic odds ratio (DOR) model. Any identified outliers were reanalyzed to validate the robustness of the results. Publication bias was assessed using Deeks’ funnel plot asymmetry test (26). Statistical significance was defined as *P* < 0.05. Clinical utility was assessed using a Fagan plot to calculate post-test probability for a given prior probability. A random-effects model was used for all pooled analyses to accommodate expected heterogeneity among studies.

## 3 Results

### 3.1 Screening and selection of articles

**Figure 1:**
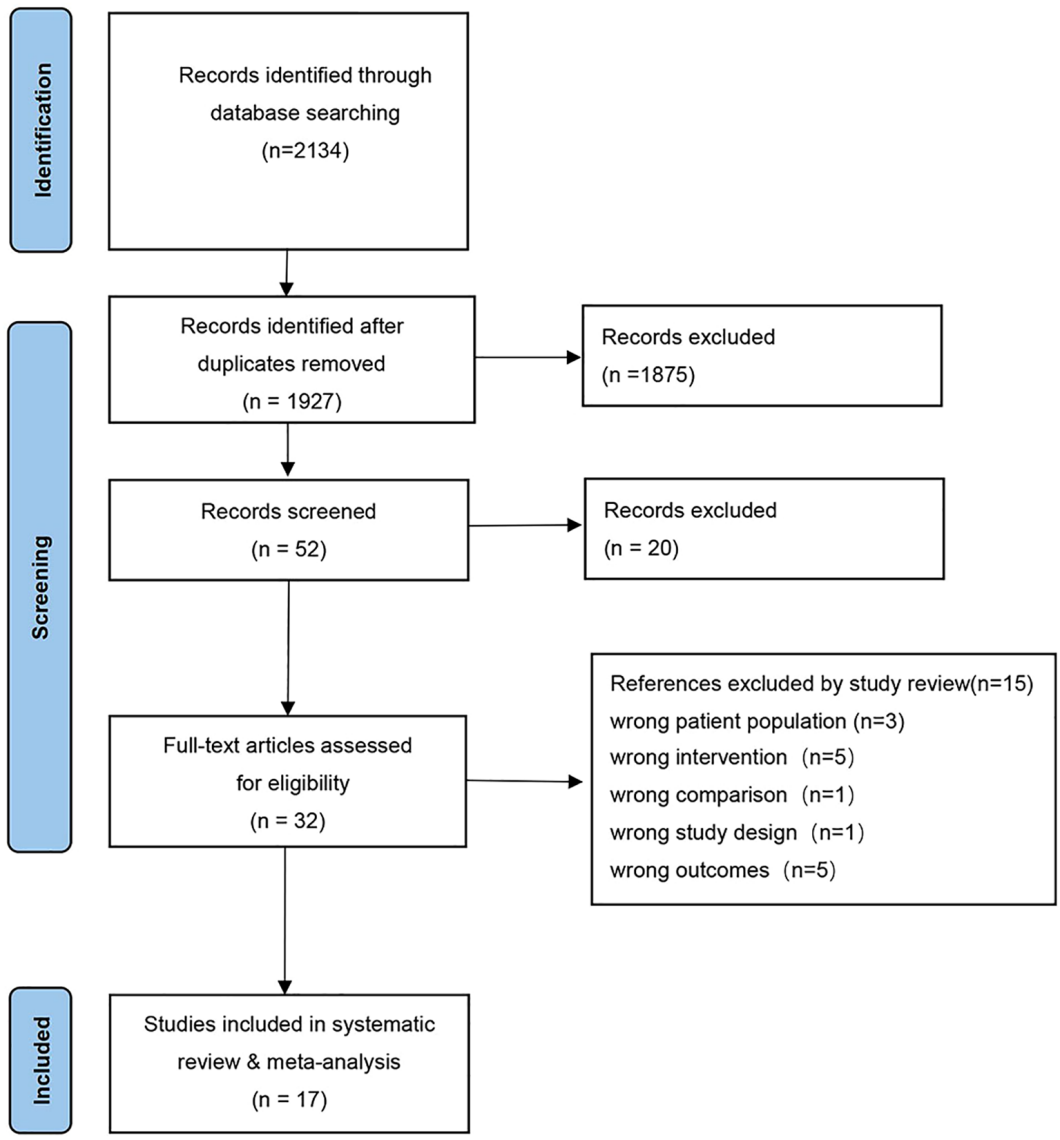
Flowchart demonstrating the process of selecting studies.

A systematic literature search, conducted according to a predefined strategy, identified 2,134 articles. After removing 207 duplicate records, 1,927 publications underwent title and abstract screening, resulting in the exclusion of 1,875 irrelevant studies. The full text of the remaining 32 papers was then assessed for eligibility. After comprehensive review, 15 articles were excluded for inconsistency with the study objectives. Ultimately, 17 articles (6, 16, 17, 27–40) conforming to the PICOS inclusion criteria were included in this study. The screening process, illustrated in the PRISMA flow diagram, is presented in **Figure 1**.

### 3.2 Study and patient characteristics

These 17 articles were published between 2022 and 2025, involving a total of 4,510 patients and 126 datasets (**Tables 1**, **2**). All included studies were conducted in China. The vast majority employed a retrospective design, with only one ambispective cohort study (38). 10 studies (6, 16, 29, 30, 32, 33, 36, 38–40) involving 48 datasets utilized multicenter data sources, while 14 studies (6, 16, 17, 27, 28, 30–32, 34–37, 39, 40), comprising 78 datasets, employed single-center data sources.

**Table 1:** General characteristics information of studies included in the systematic review.

Author	Year	No.	Training cohort			Internal validation cohorts			External validation cohorts			Pretreatment TNM	Outcome	Definition	Therpay regimen
			N+/-	Age, (year)	M/F	N+/-	Age, (year)	M/F	N+/-	Age, (year)	M/F				
She	2022	274	70/72	61.40± 9.30	121/21	30/31	61.80±8.60	53/8	48/23	63.60±7.30	68/3	I-III	MPR	≤ 10% of a viable tumor in the primary tumour bed	pembrolizumab (200 mg) or nivolumab (360 mg) combined with platinum-based chemotherapy, 2–4 cycles (3 weeks per cycle).
Liu	2023	89	40/24	NA	58/6	16/9	NA	23/2	NA	NA	NA	IB-IIIC	MPR	0-10% of viable tumor cells	chemotherapy: NA, Immunotherapy: pembrolizumab, tisleizumab, sintilimab, nivolumab, toripalimab, or camrelizumab,1–5 cycles.
Han	2024	206	63/51	NA	NA	26/24	NA	NA	7/14 10/11	63±8 63±6	16/5 21/0	IIA-IIIB	MPR	≤ 10% of a viable tumor in the primary tumour bed	at least received 2 cycles of chemoimmunotherapy.
Huang	2024	148	76/29	NA	100/5	NA	NA	NA	NA	NA	NA	II-III	MPR	≤ 10% of a viable tumor in the primary tumour bed	neoadjuvant immunochemotherapy regimen comprises 2 to 4 cycles of immunotherapy in conjunction with platinum-based chemotherapy.
Peng	2024	309	96/104	NA	194/6	NA	NA	NA	32/28 21/28	NA NA	57/3 47/2	IIa-IIIa	MPR	≤ 10% of a viable tumor in the primary tumour bed	2 to 4 cycles of chemoimmunotherapy.
Qu	2024	248	25/79	61.80 ± 9.70	90/14	24/45	60.20 ± 8.40	56/13	24/51	63.10 ± 7.40	71/4	I-III	pCR	no viable tumor cell present in primary tumors and lymph nodes	pembrolizumab or nivolumab, 2 to 4 cycles with platinum-based chemotherapy.
Wang	2024	211	87/61	65.80±7.40	136/12	37/26	64.80±7.80	58/5	NA	NA	NA	I-III	MPR	less than 10 % vital tumor tissue	at least received 2 cycles of chemoimmunotherapy.
Ye1	2024	225	43/70	60.31±6.85	105/8	NA	NA	NA	NA	NA	NA	II-III	pCR	the absence of viable tumor cells in both the tumor bed and lymph nodes	platinum-based chemotherapy in combination with pembrolizumab (200 mg), nivolumab (360 mg), durvalumab (1500 mg), nivolumab (200 mg), tislelizumab (200 mg), or camrelizumab (200 mg), at least received 2 cycles of chemoimmunotherapy
Ye2	2024	178	41/67	60.19±6.93	99/9	NA	NA	NA	23/47	60.30±9.64	56/14	II - III	pCR	absence of viable tumor cells (ypT0 and ypN0) in both the tumor bed and lymph nodes	platinum-based chemotherapy in combination with a PD-1/PD-L1 inhibitor, include pembrolizumab, nivolumab, sintilizumab, camrelizumab or tislelizumab or durvalumab, 2–4 cycles of chemoimmunotherapy.
Bao	2025	352	98/88	NA	170/16	42/38	NA	70/10	56/30	NA	69/17	IB-IIIC	MPR	less than 10% residual tumor cells	platinum-based chemotherapy in combination with a PD-1/PD-L1 inhibitor, 1 to 4 cycles.
Fan	2025	216	78/73	60.97 ± 7.11	126/25	33/32	60.97 ± 7.11	56/9	NA	NA	NA	II-III	pCR	as the absence of viable tumor cells	sintilimab, pembrolizumab, tislelizumab, camrelizumab, nivolumab, durvalumab, toripalimab, aK105/penpulimab, or anti-PD-L1 with platinum-based chemotherapy.
Gan	2025	271	45/38	60.47±10.04	63/20	17/18	63.60±7.90	30/5	59/94	61.37±8.73	129/24	IB-III	MPR	≤10% viable tumor cells	pembrolizumab (200 mg), nivolumab (360 mg), tislelizumab (200 mg), or camrelizumab (200 mg) in conjunction with platinum based chemotherapy, 2-3cycles.
Geng	2025	332	120/96	61.19 ± 7.11	192/22	NA	NA	NA	NA	NA	NA	IB-III	MPR	≤10% residual viable tumor cells in the primary tumour bed	at least received 2 cycles of chemoimmunotherapy include pembrolizumab, nivolumab, sintilimab, camrelizumab, or tislelizumab.
Han	2025	243	100/46	NA	132/14	43/18	NA	54/7	23/13	NA	34/2	I-III	MPR	less than 10% viable tumor cells in the pathological analysis of the surgical specimen	the primary preoperative chemotherapy for squamous cell carcinoma patients included intravenous paclitaxel-like and platinum-based drugs, while adenocarcinoma patients received intravenous pemetrexed combined with platinum-based drugs. The preoperative immunotherapy involved tislelizumab, pembrolizumab, camrelizumab, sintilimab (all at 200 mg) or toripalimab (240 mg), averaging two cycles.
Xiong	2025	165	43/72	NA	107/8	23/27	NA	44/6	NA	NA	NA	IB-IIIB	pCR	nonviable cancer cells in all the resected specimens	paclitaxel and platinum combined with PD-1/PD-L1 monoclonal antibody, such as tislelizumab, nivolumab, sintilimab, camrelizumab, durvalumab, toripalimab, or penpulimab, 2–4 cycles.
Ye	2025	534	120/188	60.18 ± 8.28	270/38	30/48	61.60 ± 7.39	67/11	52/96	58.66 ± 9.17	122/26	II-III	pCR	the absence of residual viable tumor cells (ypT0 and ypN0)	pembrolizumab (200 mg), nivolumab (360 mg), durvalumab (1500 mg), tislelizumab (200 mg), or camrelizumab (200 mg)—combined with platinum-based chemotherapy, 2–4 cycles.
Zheng	2025	509	195/205	NA	335/65	26/24	NA	46/4	30/29	NA	52/7	I-IIIC	MPR	no more than 10% viable tumor in the primary tumour bed	inhibitors and platinum-based chemotherapy in 21-day cycles.

F, female; M, male; MPR, major pathological response; NA, not available; No., number of patients; NSCLC, non-small cell lung cancer; pCR, pathological complete response.

**Table 2:** Radiomics-related information of studies included in the systematic review.

Author	Year	Country	Segmentation method	Feature extraction software	The best model	Classification model	Image segmentation software	Feature selection method	ICC	Study type	Reference Standard
She	2022	China	manual	3D-ShuffleNetv2x05	clinical model combined with DL model	LR and CNN	3D Slicer	DL-Based Automatic Feature Extraction	No	retrospective study	pathological tissue
Liu	2023	China	manual	NA	radiomics-clinical combined model	LR	3D Slicer	LASSO, MRMR	Yes	retrospective study	pathological tissue
Han	2024	China	semiautomatically	Pyradiomics	combines delta-radiomics features and iRECIST	LR	3D Slicer	LASSO	Yes	retrospective study	pathological tissue
Huang	2024	China	manual	PyRadiomics	combined radiomics+clinical	DT, Gaussian process, LR, RF and SVM	ITKSNAP	LASSO and 5-fold cross-validation	Yes	retrospective study	pathological tissue
Peng	2024	China	manual	NA	DL model based on ResNet50	LR and CNN	NA	LASSO and ResNet 50	No	retrospective study	pathological tissue
Qu	2024	China	manual	NA	DL model	LR	3D Slicer	Resnet-152	No	retrospective study	pathological tissue
Wang	2024	China	manual	Pyradiomics	combined radiomics+clinical	LR	ITK SNAP	LASSO, MRMR	Yes	retrospective study	pathological tissue
Ye1	2024	China	manual	NA	LUNAI-fCT	RF	ITK SNAP	FM-LCT	Yes	retrospective study	pathological tissue
Ye2	2024	China	manual	Pyradiomics	tumor internal heterogeneity habitat model	LR	ITK SNAP	LASSO	Yes	retrospective study	pathological tissue
Bao	2025	China	manual	NA	multi-timepoint short-term spatiotemporal model	SVM	ITK SNAP	LASSO, 10-fold cross-validation, SMOTE,	No	retrospective study	pathological tissue
Fan	2025	China	automatical	NA	multiregional (T + P3) radiomics model	LR, SVM, Linear SVC, DT, RF, Ada Boost, Gradient Boosting, XG Boost, Bernoulli NB, Gaussian NB, KNN, Linear Discriminant Analysis, SGD, and Multilayer Perceptron	Deepwise Multimodal Research Platform	L1 norm	No	retrospective study	pathological tissue
Gan	2025	China	manual	NA	Trans-Model	LR	ITK SNAP	Transformer fusion framework	Yes	retrospective study	pathological tissue
Geng	2025	China	manual	Pyradiomics	Transformer_GoogLeNet	ExtraTrees, WVM, MPI, MSI, MCI, and MAI	ITK SNAP	Transformer fusion framework	Yes	retrospective study	pathological tissue
Han	2025	China	manual	NA	nomogram including histology, Peri6mm and habitat signatures outperformed individual models	SVM, Extra-Trees, RF, XGBoost, LightGB, LR, AdaBoost, Naive Bayes, and Gradient Boosting	ITK-SNAP	LASSO, 10-fold cross-validation, MRMR	No	retrospective study	pathological tissue
Xiong	2025	China	manual	Pyradiomics	Rad-score and δLMR	LR	3D Slicer	LASSO	Yes	retrospective study	pathological tissue
Ye	2025	China	semiautomatically	PyRadiomics	LC-NICER prediction system	A multimodal feature-fusion based ensemble classifier	ITK-SNAP	LASSO, 5-fold cross-validation,	No	retrospective study	pathological tissue
Zheng	2025	China	manual	NA	combined model incorporating dual-phase CT scans and clinical factors	3D CNN	NA	MobileNet、ResNet-18 and One-hot	No	ambispective cohort study	pathological tissue

LR, logistic regression; CNN, convolutional neural network; CT, computed tomography; DL, deep learning; DT, decision tree; FN, and false negatives; FP, false positives; ICC, inter-class correlation coefficient; KNN, knearest neighbors; LASSO, Least Absolute Shrinkage and Selection Operator; LMR, lymphocyte-monocyte ratio; LR, logistic regression; ML, machine learning; RF, random forest; ROI, region of interest; SVM, support vector machines; TN, true negatives; TP, true positives; XGBoost, XGB, and extreme gradient boosting.

Regarding efficacy assessment, 11 studies (6, 16, 17, 27–29, 31, 34, 36, 39), involving 81 datasets, developed predictive models for MPR following neoadjuvant chemoimmunotherapy, while 6 studies (30, 32, 33, 35, 37, 40) comprising 45 datasets, predicted pCR.

Among the 17 eligible studies, different AI algorithms were employed for modeling: 8 studies (16, 29, 30, 32, 36, 38–40), incorporating 69 datasets, utilized DL algorithms, while 11 studies (6, 17, 27, 28, 30, 31, 33–37), with 57 datasets, employed ML algorithms.

In radiomics workflows, 9 studies (6, 27, 29, 32–34, 37, 39, 40), involving 74 datasets, extracted features exclusively from contrast-enhanced CT images, while the remaining studies (16, 17, 28, 30–32, 35, 36, 38) did not restrict the use of contrast-enhanced CT.

For tumor lesion segmentation, manual segmentation was commonly employed to delineate ROI within tumors. *Han* (27), *Ye* et al. (40) and *Fan* (35) et al. adopted semi-automatic and fully automatic methods, respectively. 12 studies (6, 16, 17, 27, 30, 31, 33, 34, 36–38, 40) depicted regions of interest (ROI) as 3D images, while 4 studies (28, 29, 32, 35) used 2D images. *Gan* et al. (39) used a blend of 2D and 3D images during model construction.

For radiomic feature extraction, 5 studies (16, 17, 27, 30, 37) utilized 3D Slicer software for sampling, 9 studies (6, 28, 31–34, 36, 39, 40) used ITK-SNAP software for sampling, one study (35) employed a Deepwise Multimodal Research Platform, and 2 studies (29, 38) were not referenced in the text.

LASSO was the most commonly used feature selection method. The primary classification methods employed include: LR (6, 16, 17, 27–31, 33, 35, 39), SVM (6, 28, 34, 35), RF (28, 31, 34, 36), and CNN (16, 29, 38). 15 studies (6, 16, 27–36, 38–40) standardized extracted image feature values during data processing, while 9 studies (17, 27, 28, 31–33, 36, 37, 39) used the intraclass correlation coefficient (ICC) to assess consistency among feature extractions.

Additionally, 9 studies (6, 16, 17, 27, 28, 30, 31, 37, 38), incorporating 25 datasets, combined clinical factors with radiomics features for model construction. External validation, incorporating 62 datasets, was predominantly used(n=13) (6, 16, 27–30, 32–34, 36, 38–40), while 14 studies (6, 16, 17, 27, 28, 30–32, 34–37, 39, 40), with 64 datasets, employed internal validation.

### 3.3 Quality assessment

**Figure 2:**
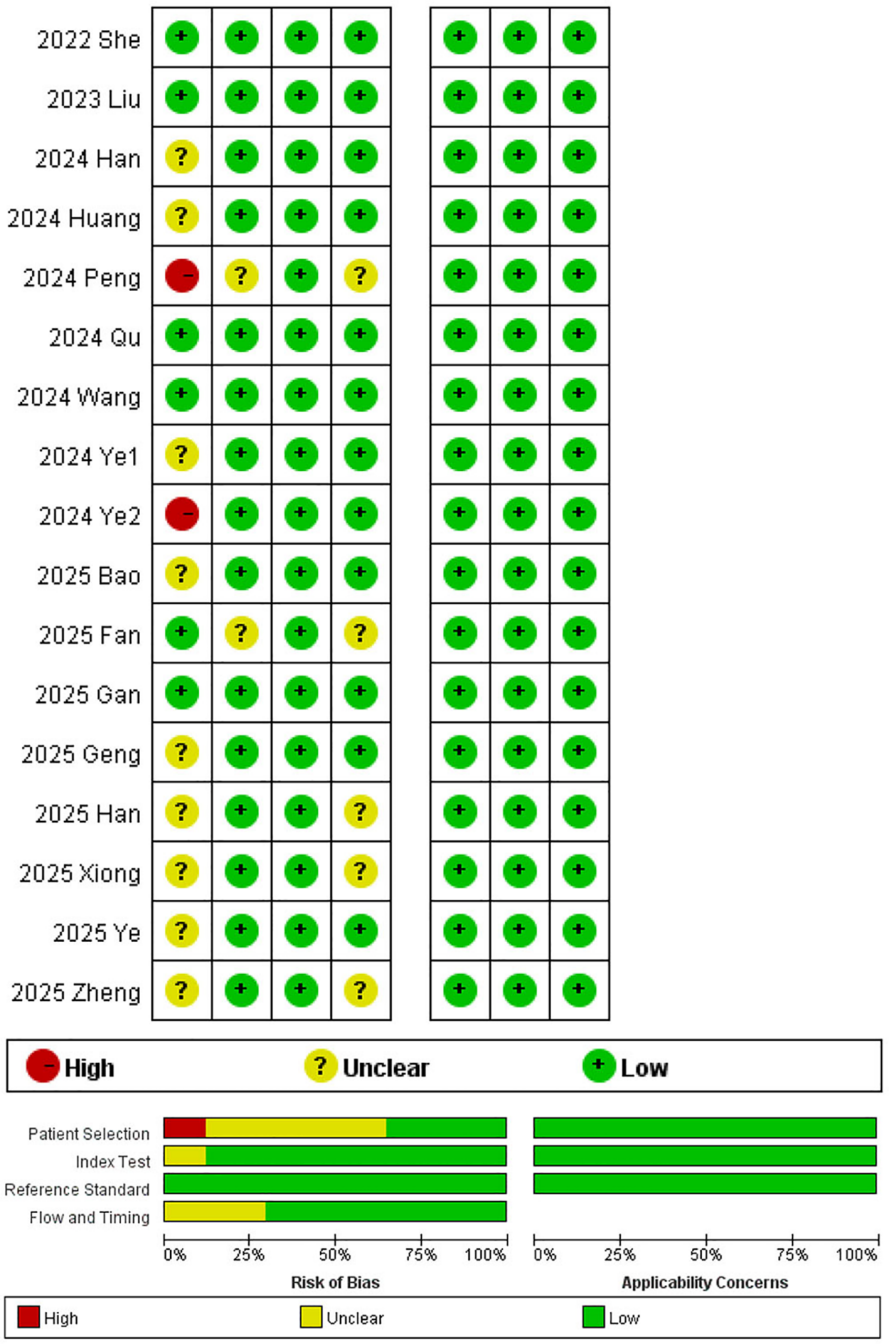
The summary of the quality assessment of the included study following QUADAS-2.

We assessed the quality of the selected studies using the QUADAS-2 tool (**Figure 2**). Overall, the methodological quality was acceptable. In the patient selection domain, 9 studies had an unclear risk of bias due to insufficient description of consecutive patient enrollment, while 2 studies showed a high risk of bias due to missing information on both consecutive enrollment and the applicable time period. In the index test domain, two studies had an unclear risk of bias due to a lack of information on blinding during the assessment process, while others showed a low risk. All studies clearly defined the reference standards. Regarding the flow and timing domain, five studies reported insufficient information on the interval between radiomics analysis and the application of the reference standard. All studies demonstrated a low risk of bias in clinical applicability.

We used the RQS 2.0 checklist shown in **Supplementary Table S3** to evaluate the quality of all radiomics studies. The scores reveal systematic methodological shortcomings. Across 9 domains, the median overall RQS was 42.31% (range 34.62%-53.57%), with an mean quality score of 42.57%. Only 1 study provided prospective data for radiomics models. Furthermore, very few studies scored well in both applicability and sustainability, and clinical Deployment.

### 3.4 Diagnostic test accuracy analysis

**Figure 3:**
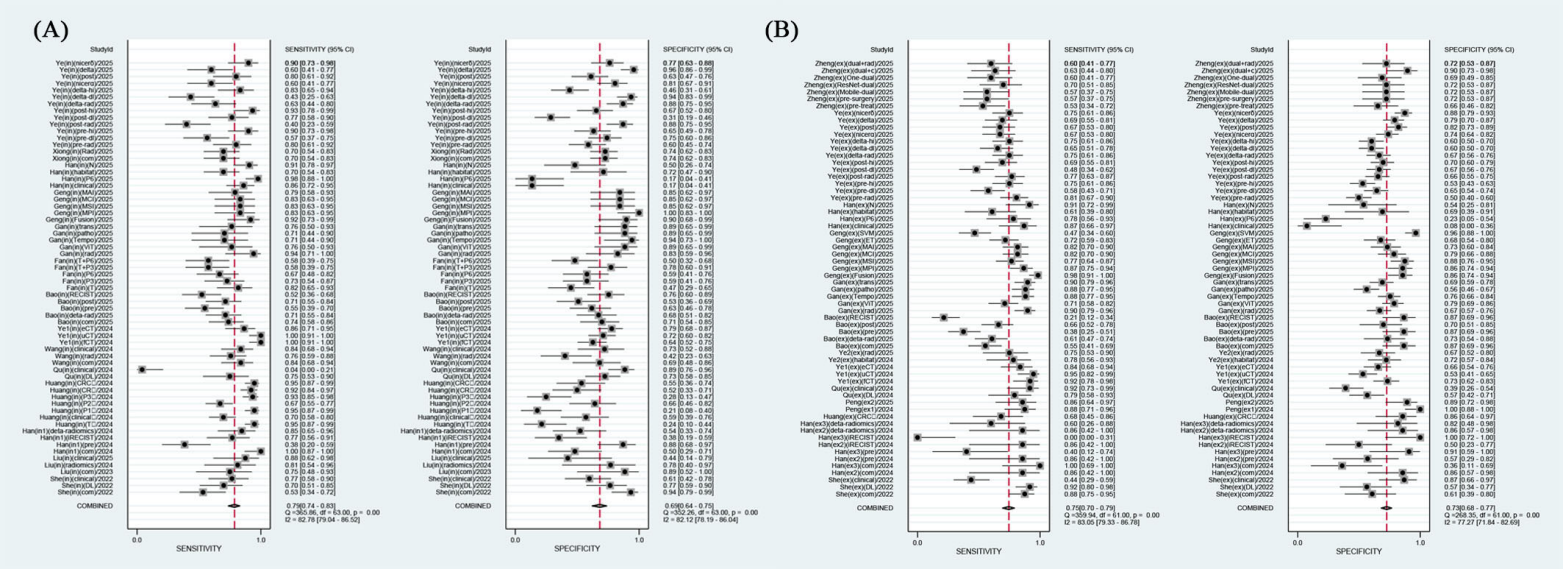
Forest plots of SEN and SPE with corresponding 95% CIs of CT-based radiomics in predicting the efficacy of neoadjuvant chemoimmunotherapy for non-small cell lung cancer on **(A)** internal validation and **(B)** external validation.

**Figure 4:**
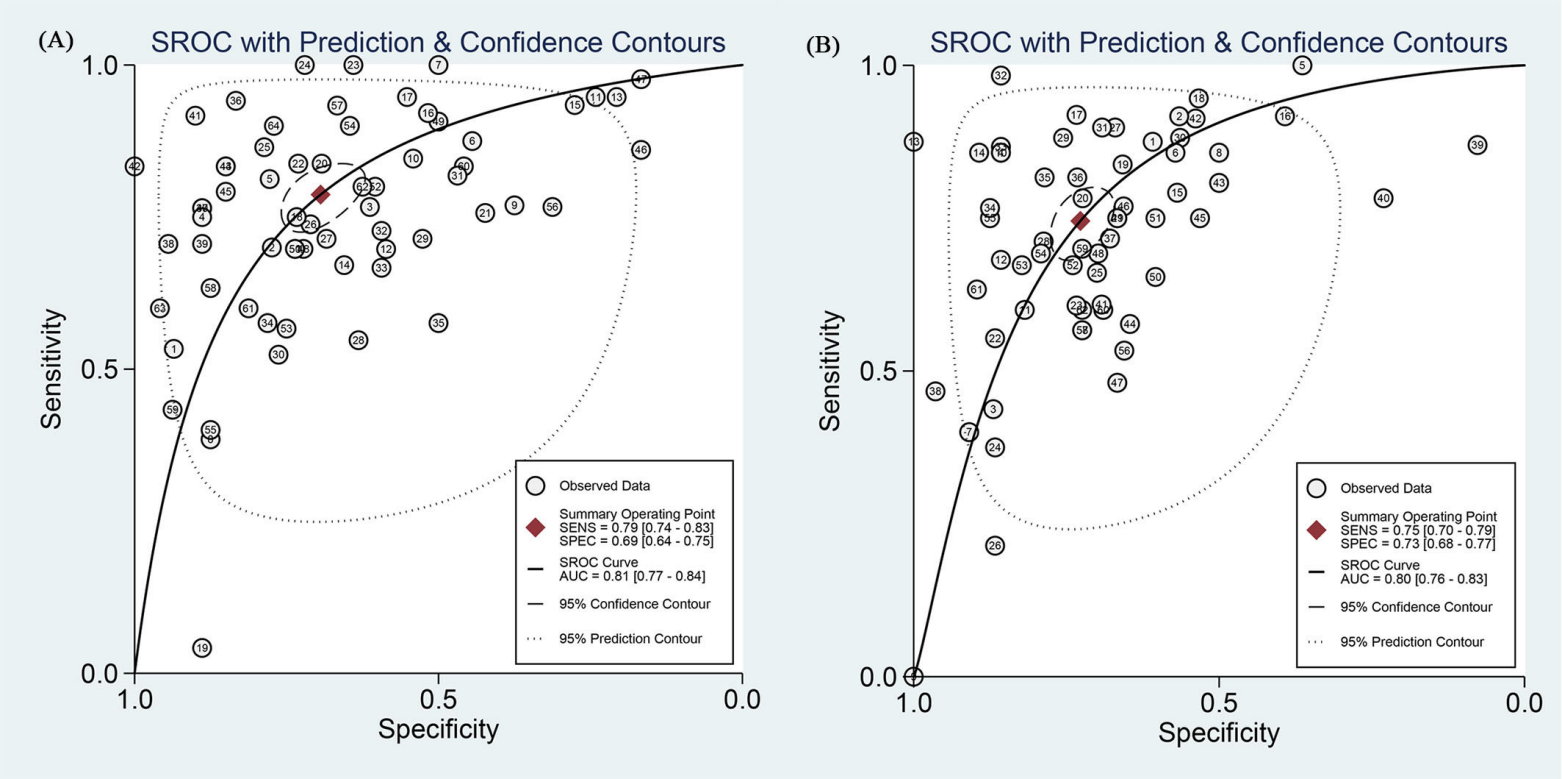
SROC curve with corresponding 95% CIs of CT-based radiomics in predicting the efficacy of neoadjuvant chemoimmunotherapy for non-small cell lung cancer on **(A)** internal validation and **(B)** external validation.

The overall radiomics model demonstrated good diagnostic performance in detecting the efficacy of neoadjuvant immunochemotherapy in NSCLC. On internal validation, the pooled AUC was 0.81 (95% CI: 0.77-0.84), sensitivity of 0.79 (95% CI: 0.74-0.83, *I^2^* = 82.78%), specificity of 0.69 (95% CI: 0.64-0.75, *I^2^* = 82.12%). The positive likelihood ratio (PLR) was 2.60 (95% CI [2.20-3.00]), the negative likelihood ratio (NLR) was 0.31 (95% CI [0.26-0.37]), and the diagnostic odds ratio (DOR) was 8 (95% CI [6-11]). On external validation, the pooled values were as follows: AUC 0.80 (95% CI: 0.76-0.83), sensitivity 0.75 (95% CI: 0.70-0.79, *I_2_* = 83.05%), specificity 0.73 (95% CI: 0.68-0.77, *I_2_* = 77.27%), PLR 2.7 (95% CI: 2.4-3.1), NLR 0.35 (95% CI: 0.30-0.41), and DOR 8 (95% CI: 6-10). The forest plot integrating sensitivity and specificity was shown in **Figure 3**, while the SROC curves from all studies were depicted in **Figure 4**.

### 3.5 Subgroup analysis

The *I^2^* statistic revealed high heterogeneity both in pooled sensitivity (*I^2^_in_* = 82.78%, *I^2^_ex_* = 83.05%) and specificity (*I^2^_in_* = 82.12%, *I^2^_ex_* = 77.27%). In internal validation, the Spearman correlation coefficient between logit(sensitivity) and logit(1-specificity) was 0.54 (*P* < 0.001). In external validation, the coefficient was 0.47 (*P* = 0.001), indicating a moderate threshold effect across studies. Subgroup analyses were conducted to identify potential sources of heterogeneity and threshold effect, with groups adjusted as shown in **Tables 3** and **4**. The meta-regression analyses indicated that the factor of different AI algorithms, imaging method, efficacy evaluation, ICC, RQS and ROI may contribute to sources of heterogeneity.

**Table 3:** Results of meta-regression and subgroup analyses of radiological model in internal datasets.

Characteristic	Category	Number of datasets	Sensitivity (95% CI)	*P*1	Specificity (95% CI)	*P*2	*P*3
Multi-center	Yes	13	0.73 (0.62-0.84)	< 0.001	0.75 (0.65-0.85)	0.20	0.43
	No	51	0.80 (0.75-0.85)		0.68 (0.62-0.74)		
AI algorithms	DL	30	0.79 (0.73-0.85)	< 0.001	0.79 (0.74-0.85)	0.96	< 0.001
	ML	34	0.79 (0.73-0.84)		0.58 (0.51-0.65)		
Imaging method	contrast-enhanced	34	0.76 (0.70-0.83)	< 0.001	0.71 (0.64-0.78)	0.05	0.53
	CT	30	0.81 (0.75-0.87)		0.68 (0.59-0.76)		
Efficacy Evaluation	MPR	39	0.82 (0.77-0.86)	0.02	0.67 (0.60-0.74)	< 0.001	0.22
	pCR	25	0.74 (0.66-0.81)		0.73 (0.65-0.80)		
Normalized	Yes	59	0.79 (0.74-0.83)	0.26	0.69 (0.64-0.75)	0.27	0.95
	No	5	0.77 (0.61-0.94)		0.72 (0.53-0.91)		
Combined clinical parameters	Yes	14	0.77 (0.61-0.87)	< 0.001	0.68 (0.56-0.80)	0.06	0.79
	No	50	0.79 (0.74-0.84)		0.70 (0.64-0.76)		
ICC	Yes	32	0.85 (0.80-0.89)	0.04	0.70 (0.63-0.78)	0.03	< 0.001
	No	32	0.71 (0.64-0.78)		0.69 (0.61-0.76)		
RQS	>41.07%	45	0.78 (0.73-0.84)	< 0.001	0.71 (0.65-0.78)	0.24	0.46
	≤41.07%	19	0.79 (0.71-0.87)		0.64 (0.54-0.75)		
ROI	3D	44	0.76 (0.70-0.81)	< 0.001	0.71 (0.66-0.77)	0.87	< 0.001
	2D	15	0.86 (0.80-0.92)		0.55 (0.44-0.67)		

MPR, major pathological response; pCR, pathological complete response; P_1_, represents the p-value for the association between the subgroup variable and the sensitivity of the diagnostic test; P_2_, denotes the p-value for the association between the subgroup variable and the diagnostic test's specificity; P_3_ denotes the p-value for the association between the subgroup variable and the diagnostic test's Diagnostic Odds Ratio (DOR); RQS, Radiomics Quality Score; ICC, Intraclass correlation coefficient.

**Table 4:** Results of meta-regression and subgroup analyses of radiological model in external datasets.

Characteristic	Category	Number of datasets	Sensitivity (95% CI)	*P*1	Specificity (95% CI)	*P*2	*P*3
Multi-center	Yes	35	0.80 (0.75-0.85)	0.18	0.72 (0.67-0.78)	< 0.001	< 0.001
	No	27	0.65 (0.57-0.73)		0.74 (0.68-0.80)		
AI algorithms	DL	39	0.77 (0.72-0.82)	0.08	0.73 (0.68-0.78)	< 0.001	0.12
	ML	23	0.68 (0.59-0.77)		0.72 (0.65-0.79)		
Imaging method	contrast-enhanced	40	0.73 (0.67-0.79)	< 0.001	0.71 (0.66-0.76)	< 0.001	0.28
	CT	22	0.77 (0.70-0.84)		0.75 (0.69-0.81)		
Efficacy Evaluation	MPR	42	0.74 (0.68-0.80)	< 0.001	0.76 (0.72-0.81)	0.03	0.05
	pCR	20	0.76 (0.69-0.84)		0.66 (0.59-0.73)		
Combined clinical parameters	Yes	11	0.80 (0.70-0.90)	0.14	0.66 (0.54-0.78)	< 0.001	0.36
	No	51	0.73 (0.68-0.79)		0.74 (0.70-0.78)		
ICC	Yes	26	0.81 (0.76-0.87)	0.05	0.77 (0.71-0.82)	0.01	< 0.001
	No	36	0.70 (0.63-0.76)		0.70 (0.64-0.76)		
RQS	>41.07%	49	0.71 (0.66-0.76)	< 0.001	0.75 (0.71-0.79)	0.30	0.01
	≤41.07%	13	0.86 (0.79-0.92)		0.63 (0.53-0.73)		
ROI	3D	51	0.71 (0.66-0.76)	< 0.001	0.72 (0.67-0.77)	0.01	< 0.001
	2D	6	0.87 (0.79-0.96)		0.80 (0.68-0.91)		

MPR, major pathological response; pCR, pathological complete response; P_1_, represents the p-value for the association between the subgroup variable and the sensitivity of the diagnostic test; P_2_, denotes the p-value for the association between the subgroup variable and the diagnostic test's specificity; P_3_, denotes the p-value for the association between the subgroup variable and the diagnostic test's Diagnostic Odds Ratio (DOR); RQS, Radiomics Quality Score; ICC, Intraclass correlation coefficient.

In terms of data sources on internal validation, multi-center studies (n=13) demonstrated lower sensitivity (0.73 [95% CI, 0.62-0.84] compared to single-center studies (n=51, 0.80, 95% CI: 0.75-0.85; *P* < 0.001). In external validation, the specificity of multicenter studies (n=35) was lower than that of single-center studies (n=27, 0.74, 95% CI: 0.68-0.80; *P* < 0.001).

Regarding AI algorithms in internal validation, models using DL algorithms (n=30) exhibited higher diagnostic sensitivity (0.79 [95% CI, 0.73-0.85] than those using ML algorithms (n=34, 0.79, 95% CI: 0.73-0.84; *P* < 0.001). However, their effect on specificity was not significant (*P* = 0.96), although DL showed a higher point estimate for specificity. In external validation, this pattern shifted: DL models (n=39) demonstrated superior specificity (0.73 [95% CI,0.68-0.78]) (*P* < 001) compared to ML models (n=23, 0.72 [95% CI,0.65-0.79]). Sensitivity also showed a trend toward superiority for DL (0.77 vs 0.68, *P* = 0.08).

By imaging method in internal validation, models constructed using enhanced CT alone (n=34) demonstrated lower sensitivity (0.76 [95% CI, 0.70-0.83] vs 0.81 [95% CI, 0.75-0.87]) (*P* < 0.001). This trend persists in the external validation datasets (0.73 [95% CI, 0.67-0.79] vs 0.77 [95% CI, 0.70-0.84]) (*P* < 0.001). Furthermore, the specificity of enhanced CT (n=40) was also significantly reduced (0.71 [95% CI, 0.66-0.76] vs 0.75 [95% CI, 0.69-0.81]) (*P* < 0.001).

Regarding efficacy assessment in internal validation, the model constructed to predict MPR(n=39) following neoadjuvant chemotherapy combined with immunotherapy demonstrated superior sensitivity (0.82 [95% CI, 0.77-0.86] vs (0.74 [95% CI, 0.66-0.81] (*P* = 0.02) but lower specificity (0.67 [95% CI, 0.60-0.74] vs 0.73 [95% CI, 0.65-0.80]) (*P* < 0.001) over the model predicting pCR (n=25). In external validation, MPR-predicting models (n=42) demonstrated slightly lower sensitivity (0.74 [95% CI, 0.68-0.80] vs 0.76 [95% CI, 0.69-0.84]) (*P* < 0.001), but higher specificity (0.76 [95% CI, 0.72-0.81] vs 0.66 [95% CI, 0.59-0.73]) (*P* = 0.03) than pCR-predicting models.

Studies combining radiomics with clinical factors demonstrated lower sensitivity in internal validation (0.77, 95% CI: 0.61-0.87 vs. 0.79, 95% CI: 0.74-0.84; *P* < 0.001) and lower specificity in external validation (0.66, 95% CI: 0.54-0.78 vs. 0.74, 95% CI: 0.70-0.78; P < 0.001).

Imaging features selected based on ICC demonstrated significantly improved sensitivity and specificity in both internal and external validation datasets. In internal validation, sensitivity was 0.85 (95% CI: 0.80-0.89) vs 0.71 (95% CI: 0.64-0.78) (*P* = 0.04), and specificity was 0.70 (95% CI: 0.63-0.78) vs 0.69 (95% CI: 0.61-0.76) (*P* = 0.03). In external validation, sensitivity was 0.81 (95% CI: 0.76-0.87) vs 0.70 (95% CI: 0.63-0.76) (*P* = 0.05), and specificity was 0.77 (95% CI: 0.71-0.82) vs 0.70 (95% CI: 0.64-0.76) (*P* = 0.01).

In internal validation, the pooled sensitivity for studies with an RQS score over 41.07% was slightly lower (0.78, 95% CI: 0.73-0.84) compared to those with a lower score (0.79, 95% CI: 0.71-0.87; *P* < 0.001). This trend was more pronounced in external validation, where higher RQS scores were associated with lower sensitivity (0.71, 95% CI: 0.66-0.76 vs. 0.86, 95% CI: 0.79-0.92; *P* < 0.001), indicating that diagnostic sensitivity decreased as the RQS score increased.

In internal validation, models using 2D ROIs demonstrated higher sensitivity (0.86 [95% CI, 0.80-0.92]) than those using 3D ROIs (0.76 [95% CI, 0.70-0.81]). The diagnostic accuracy of 2D ROIs was further supported in external validation (sensitivity: 0.87, 95% CI: 0.79-0.96; specificity: 0.80, 95% CI: 0.68-0.91).

### 3.6 Sensitivity analyses

**Figure 5:**
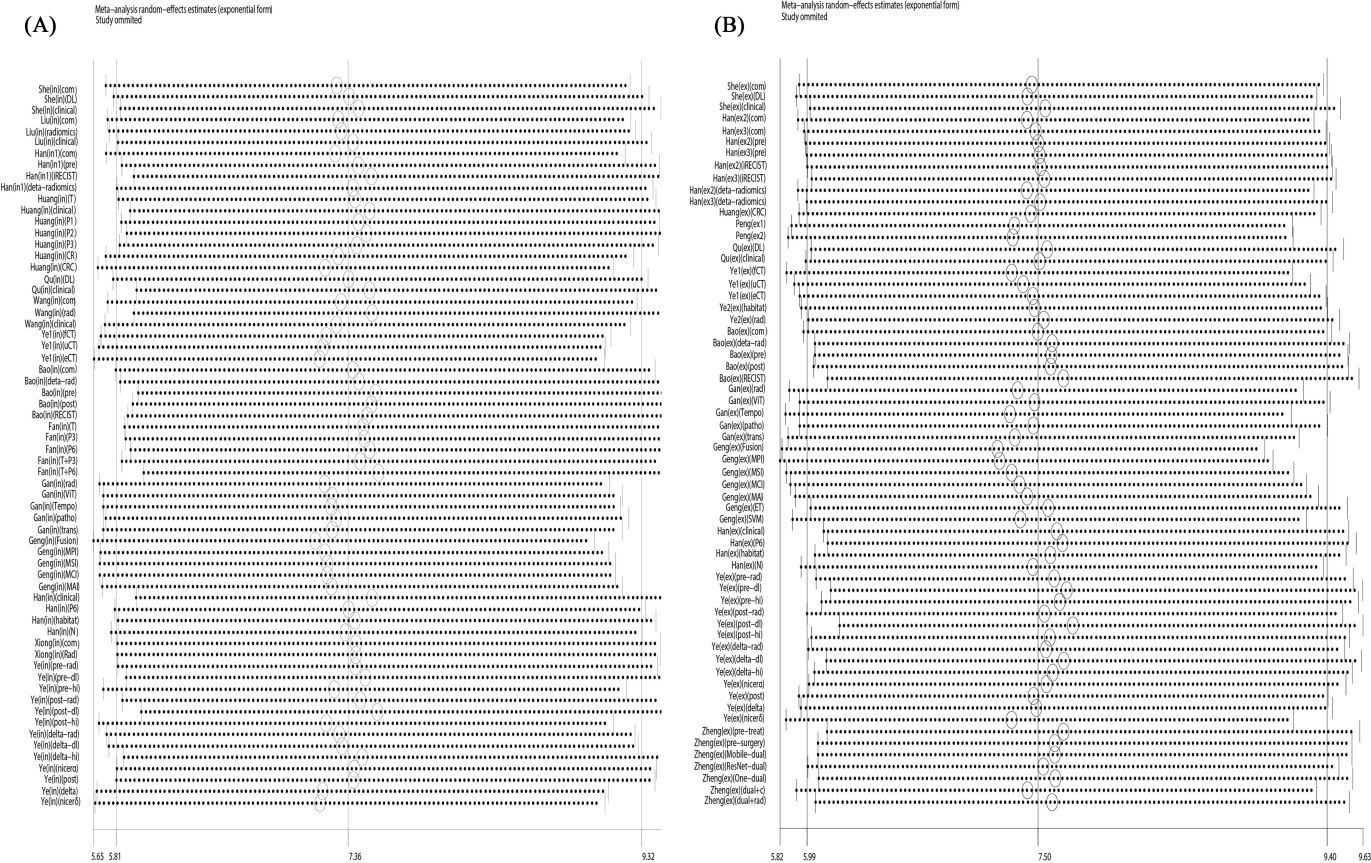
The figure for sensitivity analysis was calculated using the stepwise rejection method on **(A)** internal validation and **(B)** external validation.

As shown in **Figure 5**, sensitivity analysis indicates no significant changes after each systematic exclusion of a study.

### 3.7 Publication bias

**Figure 6:**
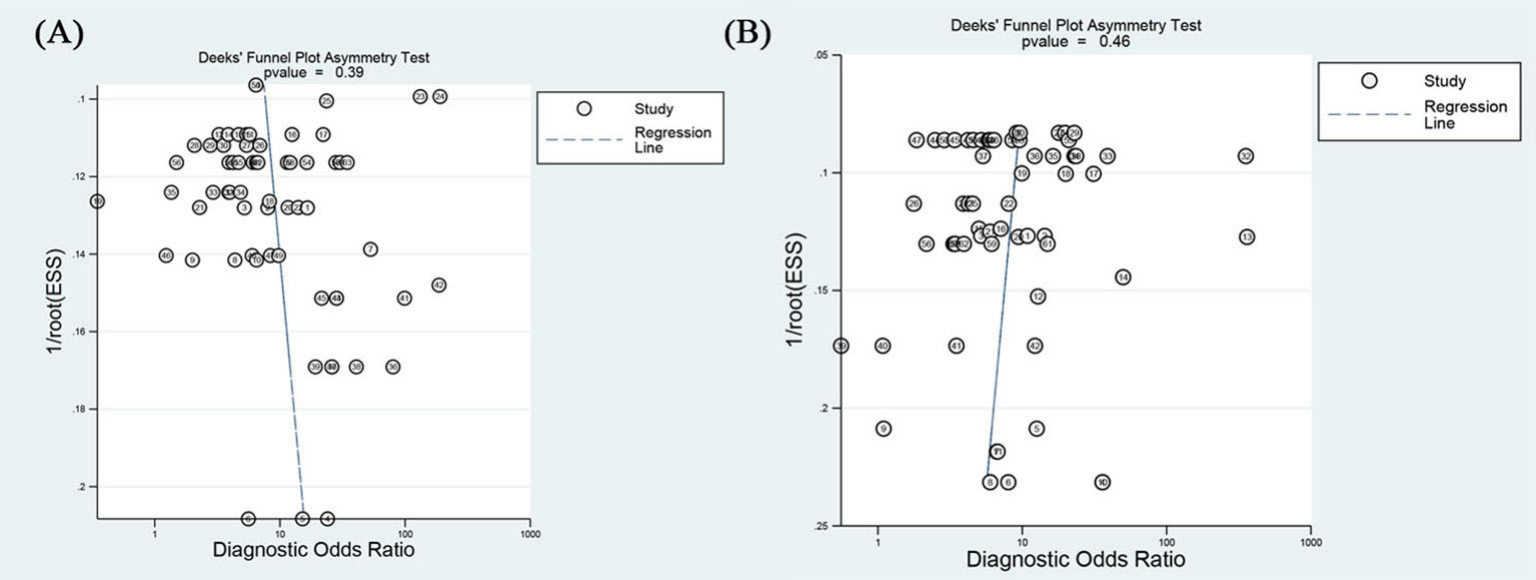
Funnel plot based on radiomics model in predicting the efficacy of neoadjuvant chemoimmunotherapy for non-small cell lung cancer on **(A)** internal validation and **(B)** external validation.

To assess publication bias, a Deeks funnel plot asymmetry test was conducted (**Figure 6**). The results showed no substantial evidence of publication bias both on internal (*P_in_* = 0.39), and external validation (*P_ex_* = 0.46).

### 3.8 Clinical utility

**Figure 7:**
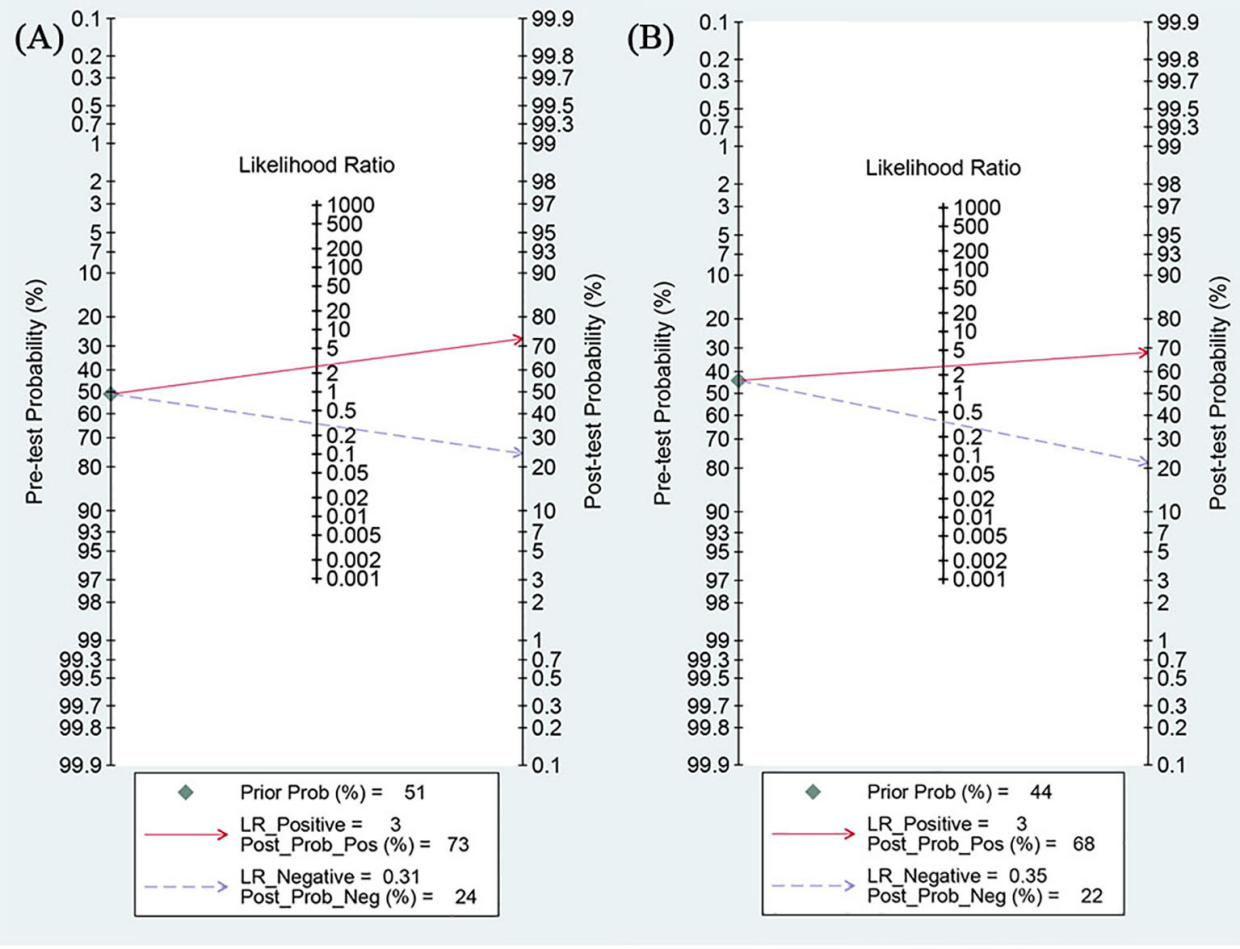
The Fagan nomogram of the radiomics model in predicting the efficacy of neoadjuvant chemoimmunotherapy for non-small cell lung cancer on **(A)** internal validation and **(B)** external validation.

Based on Fagan’s histogram analysis, in internal validation, applying a radiomics model with a pre-test probability of 51% and a positive likelihood ratio (PLR) of 3, the predicted probability of NSCLC patients achieving MPR or pCR after neoadjuvant chemotherapy and immunotherapy can be elevated to 73%. Conversely, under the same model framework, a negative likelihood ratio (NLR) reduces this positive a posteriori probability to 24%. In external validation, a model with a pre-test probability of 44% and a PLR of 3 elevated the predicted probability to 68%. Conversely, an NLRof 0.35 reduces the posterior positive probability to 22% (**Figure 7**).

## 4 Discussion

Neoadjuvant chemotherapy is an emerging adjuvant therapy for lung cancer, primarily aimed at suppressing tumor immune escape, activating the body’s immune response, and eliminating tumor cells (41). Compared to traditional neoadjuvant chemotherapy, neoadjuvant chemotherapy enhances the feasibility of curative surgery and improves long-term survival, representing a significant advancement in lung cancer treatment (15, 42–44). However, despite significant progress, a substantial proportion of patients fail to benefit from this approach (45).

Accurate prediction of treatment response is critical for stratifying and selecting those who will derive the greatest benefit from neoadjuvant chemoimmunotherapy while avoiding immune-related adverse events (irAEs). In clinical practice, CT-based RECIST assessment is the standard method for monitoring systemic therapy responses (46). However, due to the specific tumor response patterns induced by immunotherapy—such as pseudoprogression, hyperprogression, and delayed response—pathological responses in 41% to 45% of NSCLC patients undergoing neoadjuvant therapy do not correlate with imaging evaluations (47).

Radiomics technology can extract features from lung cancer CT images to establish predictive models for evaluating the efficacy of novel anticancer drugs in lung cancer, enabling early identification of drug resistance and providing guidance for optimizing and adjusting treatment regimens (48). Recent studies have confirmed the effectiveness of radiomics in predicting pathological response to neoadjuvant chemoimmunotherapy in resectable NSCLC patients. For instance, a meta-analysis demonstrated that ΔSUVmax serves as a reliable early predictor of MPR to neoadjuvant immunochemotherapy in NSCLC (49). CT holds broader applicability in NSCLC and remains an indispensable modality for evaluating the efficacy of neoadjuvant chemoimmunotherapy. However, meta-analyses examining CT-based radiomics for predicting the efficacy of neoadjuvant chemoimmunotherapy in NSCLC are currently absent.

### 4.1 Key findings

This study conducted a systematic review and meta-analysis of 17 studies establishing CT-based predictive models for the efficacy of neoadjuvant chemoimmunotherapy in NSCLC. To our knowledge, this represents the first comprehensive evaluation of radiomics techniques applied to this specific treatment context. The pooled results indicate that these models demonstrate good performance in predicting neoadjuvant treatment efficacy (SROCin = 0.81 [0.77-0.84], SROCex = 0.80 [0.76-0.83]). The consistency between internal validation models and external validation models reflects their robustness. Utilizing radiomics to predict the efficacy of neoadjuvant chemoimmunotherapy in NSCLC may provide valuable guidance for clinical decision-making.

We observed heterogeneity in the pooled sensitivity (*I*^2^in = 82.78%, *I*^2^ex = 83.05%) and specificity (*I*^2^in = 82.12%, *I*^2^ex = 77.27%). This may be attributed to methodological differences and variations in radiomics workflows across studies, as shown in **Table 3**. Several factors—including the integration of clinical parameters, choice of AI algorithm, efficacy evaluation metric, use of ICC, RQS score, and ROI dimensions—collectively exerted statistically significant effects on the results and contributed to the observed variability.

A sensitivity analysis demonstrated that excluding any single study from the pool of 17 did not significantly impact the pooled sensitivity and specificity, confirming the robustness of our findings. This result enhances the reliability of our conclusions regarding the effectiveness of radiomics in predicting neoadjuvant chemoimmunotherapy efficacy. Regarding clinical application, in internal validation, a positive radiomics model result (PLR = 3) increased the probability of an effective response from a pre-test probability of 51% to a post-test probability of 73%. Conversely, a negative model result (NLR = 0.31) reduced the probability of an effective response to 24%. In external validation, a positive result (PLR = 3) increased the probability from a pre-test probability of 44% to 68%, while a negative result (NLR = 0.35) reduced it to 22%.

### 4.2 MPR and pCR: complementary predictive values

Among numerous neoadjuvant immunotherapy trials for solid tumors, pathological response has been widely recognized as a surrogate endpoint (50). Unlike other cancers such as breast and bladder cancer, the rate of complete pathological response (pCR) following chemotherapy for NSCLC remains relatively low, ranging from 9% to 63%. Conversely, 27% to 86% of NSCLC patients achieve MPR following neoadjuvant immunotherapy (51). This suggests that setting MPR as a predictive endpoint yields a more balanced training sample and identifies nearly twice as many patients likely to benefit from neoadjuvant therapy compared to pCR. The clinical significance of MPR prediction lies in its strong association with survival benefit and its utility as a surrogate endpoint for assessing treatment efficacy in thoracic oncology (52).

A key strength of this study is the consistency definition of critical pathological endpoints (pCR and MPR) across the literature, which enhances the comparability of results and the reliability of the pooled estimates. Our meta-regression analysis revealed a significant trade-off between models based on these two endpoints, though the specific advantage was dependent on the validation setting. In internal validation, MPR-based models demonstrated superior sensitivity, while pCR-based models showed greater specificity. Interestingly, this pattern shifted in external validation, where MPR-based models exhibited stronger specificity, whereas pCR-based models tended toward higher sensitivity. This dichotomy and its context-dependence likely stem from inherent differences in their pathological definitions and the challenges of generalizing predictive models. MPR, defined as a continuum of residual viable tumor (≤10%), captures progressive biological responses that may be more continuous and thus more susceptible to detection by evolving radiomics features during treatment. Conversely, pCR represents an absolute state of no viable tumor cells. While this stringent criterion yields highly specific signals, it may fail to detect microscopic residual disease on imaging, resulting in reduced sensitivity. The observed performance reversal in external datasets may reflect greater heterogeneity in imaging protocols and case mix, which differentially impacts the detection thresholds for a graded (MPR) versus a binary (pCR) outcome.

Notably, the superior specificity of the pCR-based model in internal validation highlights its unique value as a definitive “all-or-nothing” endpoint. Achieving pCR serves as a potent prognostic indicator, frequently associated with the deepest molecular response and optimal long-term survival rates. Consequently, its precise identification (i.e., high specificity) is crucial for dose-reduction trials or selecting candidates for curative intent. For thoracic surgeons, preoperative MPR prediction aids risk stratification, informing decisions on optimal surgical timing and adjuvant therapy planning. This non-invasive approach reduces reliance on invasive biopsies, aligning with the growing demand for personalized treatment optimization in locally advanced NSCLC. By accurately reflecting tumor response to therapy, MPR is regarded as a key prognostic marker for resectable NSCLC. Studies demonstrate a significant correlation between MPR and long-term overall survival (OS) in NSCLC patients receiving neoadjuvant chemotherapy and immunotherapy (53). This underscores MPR’s utility as a survival surrogate endpoint and tool for evaluating neoadjuvant efficacy in clinical trials.

In clinical decision-making, prioritizing MPR or pCR as a predictive endpoint should align with specific treatment objectives: If the goal is to maximize identification of all potential responders to avoid undertreatment, the more sensitive MPR model should be preferred. Conversely, when the goal is to select patients most likely to achieve deep, curative responses for intensive monitoring or dose-reduction strategies, the higher-specificity pCR model is more appropriate. Ultimately, integrating both endpoints through sequential or combined modeling-assessing tumor response comprehensively (MPR) while confirming superior efficacy (pCR) —would best balance the needs of precision treatment.

### 4.3 Advantages of deep learning algorithms

Our subgroup analysis demonstrated that prediction models constructed using DL algorithms outperformed those based on ML algorithms. This finding is consistent with a previous meta-analysis of CT-based radiomics models predicting air-space spread in lung cancer, where the DL subgroup demonstrated higher pooled sensitivity (0.87 vs. 0.81, *P* < 0.001) and comparable pooled specificity (0.77 vs. 0.75, *P* < 0.001) (54).

As a complex subset of ML, has the potential to uncover subtle imaging features not readily apparent to the human eye, providing complementary information for predicting neoadjuvant immunotherapy efficacy (55). Its application in tumor radiomics analysis plays an increasingly vital role in diagnosis, treatment decision-making, and prognosis (56, 57). For instance, *Lin* et al. (58) conducted a retrospective analysis of clinical and imaging data from 62 NSCLC patients undergoing neoadjuvant immunotherapy. They extracted radiomic and DL features from lung cancer lesions to construct an integrated model combining clinical characteristics, radiomic features, and DL functions for accurate response prediction. *She* et al. (16) developed a DL model to predict MPR, achieving an AUC of 0.72 in an external validation cohort.

The primary limitation of DL models is their lack of interpretability, posing a persistent challenge for deploying this “black-box” technology in clinical practice. However, recent research has begun to address this issue by visualizing the image regions the network deems important, thereby elucidating the predictive process of these models (16, 29). *Peng* et al. recruited 309 LUSC patients from multiple medical institutions and developed a ResNet-50 model. Using the Grad-CAM method to visualize CT images, they identified critical regions for predicting MPR. The DL model demonstrated excellent predictive accuracy, achieving an area under the AUC of 0.95 (95% CI: 0.98-1.00) and a sensitivity of 0.90 (95% CI: 0.81-0.98) (29).

In summary, AI-based radiomics and DL models demonstrate significant potential in predicting the efficacy of neoadjuvant chemoimmunotherapy treatment for lung cancer. These models can assist clinicians in early identification of patient response and resistance, thereby providing valuable insights for optimizing and transforming immunotherapy treatment plans.

### 4.4 Optimization of radiology workflow processes

To mitigate the risk of overestimating model performance, this meta-analysis exclusively utilizes validated data from radiomics studies, synthesizing internal and external validation results separately. Currently, most developed radiomics models assess predictive performance through internal validation. Literature indicates that external validation is recommended for datasets exceeding 50 samples, while revalidation methods are preferred for smaller datasets (59). In this study, three investigations employed cohorts recruited at different time points from a unified research center as external validation sets. The AUC values obtained from external validation were generally lower than those from internal validation or training cohorts, a pattern consistent with findings in previous studies.

In radiomics model development, ICC is used to assess the repeatability and robustness of imaging features extracted from tumor lesions across different individuals, segmentation methods, and time points. This assessment encompasses inter-observer and intra-observer variability. Previous reviews indicate that most imaging features exhibit high robustness to inter-observer or intra-observer variability. Consistent with these findings, our study suggests that using features without ICC filtering may lead to reduced diagnostic sensitivity and specificity. Nevertheless, numerous sources of heterogeneity exist for ICC values, including imaging scanner parameters, imaging resolution, tumor segmentation methods, feature extraction software, etc. Furthermore, threshold settings remain unstandardized, precluding any quantitative evaluation of ICC to date (60). Within the internal dataset, the 2D model demonstrated high sensitivity (0.86) but low specificity (0.55). This suggests the model may enhance detection capabilities by overfitting to local features of specific two-dimensional slices in the training data. Conversely, this also led to misclassification of non-target patterns, indicating overfitting. However, in the external dataset, a small number of 2D models (n=6) reported both high sensitivity (0.87) and specificity (0.80). This discrepancy may stem from the 2D analysis’s heavy reliance on the operator’s selection of the “most representative “ slice. During internal validation, this selective process may have optimized for sensitivity at the cost of specificity. In certain ideal external scenarios, however, a similarly well-chosen representative slice may prove equally effective, resulting in better-balanced performance.

Among the 17 studies included in this analysis, the average RQS score was 42.57%, indicating significant room for improvement. The original RQS framework established benchmarks for evaluating the quality and reporting of radiomics research. However, advancements in AI have introduced new complexities to the field, raising the need to address challenges such as fairness, generalizability, accessibility, and interpretability. RQS 2.0 accounts for this evolution by distinguishing between supervised learning and DL approaches. Through its integration with the Radiomics Reporting Layer (RRL), it provides a more comprehensive framework that not only assesses the scientific quality of radiomics studies but also establishes checkpoints to facilitate their effective translation into clinical practice (23). In a subgroup analysis stratified by the median RQS score (41.07%), studies with higher scores demonstrated a lower pooled diagnostic sensitivity (internal validation: 0.78 [95% CI: 0.73-0.84]; external validation: 0.71 [95% CI: 0.66-0.76]) but a trend toward improved specificity (internal validation: 0.71 [95% CI: 0.65-0.78]; external validation: 0.75 [95% CI: 0.71-0.79]). It’s potentially attributable to inter-rater variability during RQS scoring and challenges in RQS scoring reproducibility (61).Furthermore, few studies scored in the areas of Prospective Validity, Applicability and Sustainability, and Clinical Deployment. This highlights directions for improvement in future research.

### 4.5 Limitation

This study has several limitations that warrant careful consideration when interpreting the findings. First, all included studies were conducted in China, which raises concerns regarding the generalizability of the results—a concept known as spectrum bias. The external validity of our findings to non-Chinese populations remains unproven. Spectrum effects may arise from differences in the mix of disease stages, the proportion of squamous cell carcinoma versus adenocarcinoma (which have distinct biological behaviors and may respond differently to chemoimmunotherapy), as well as variations in regional treatment protocols and healthcare systems. Therefore, the predictive performance of the summarized radiomics models requires rigorous validation in multinational, prospective cohorts before they can be applied to other ethnic or geographic populations.

Second, significant technical heterogeneity existed across the studies, representing a fundamental challenge in radiomics research. Variations in CT scanner manufacturers, models, acquisition parameters (e.g., slice thickness, tube voltage), and reconstruction kernels directly affect the extraction and reproducibility of radiomics features. This scanner and protocol heterogeneity likely introduces substantial noise into the pooled analysis and may degrade model performance and robustness when applied to external datasets using different equipment. Future multicenter studies should employ standardized imaging protocols or advanced harmonization techniques (e.g., ComBat) to mitigate this issue.

Third, clinical and methodological heterogeneity was evident among the included studies. The use of ambispective or retrospective designs introduces risks of selection and verification bias. While our meta-regression attempted to explore some of these factors, these analyses were exploratory and limited by the number of available studies. Future research should prioritize prospective designs with prespecified, standardized protocols for patient stratification, treatment, and outcome assessment to develop more reliable and subtype-specific predictive models.

## 5 Conclusion

Radiomics technology demonstrates strong diagnostic capability in predicting the efficacy of neoadjuvant chemoimmunotherapy in NSCLC. However, current limitations associated with this technology may restrict its direct clinical application. Further comprehensive studies are needed to validate the findings of this research and promote the clinical application of AI and imaging in this field in the future.

## Data Availability

The datasets presented in this study can be found in online repositories. The names of the repository/repositories and accession number(s) can be found in the article/**Supplementary Material**.
