# Supplementary material for: CT-based radiomics in predicting the efficacy of preoperative neoadjuvant chemoimmunotherapy for non-small cell lung cancer: a systematic review and meta-analysis
Source: Front Immunol. 2026 Feb 10;17:1753166. doi: 10.3389/fimmu.2026.1753166 (PMC12929539; doi:10.3389/fimmu.2026.1753166)
Supplement: Supplementary file 1 [file Table1.docx]

**Supplementary Table 1. PRISMA checklist of this Meta-analysis.**

| **Section and Topic** | **Item #** | **Checklist item** | **Location where item is reported** |
| --- | --- | --- | --- |
| **TITLE** | | |  |
| Title | 1 | Identify the report as a systematic review. | 1 |
| **ABSTRACT** | | |  |
| Abstract | 2 | See the PRISMA 2020 for Abstracts checklist. | 1 |
| **INTRODUCTION** | | |  |
| Rationale | 3 | Describe the rationale for the review in the context of existing knowledge. | 2-3 |
| Objectives | 4 | Provide an explicit statement of the objective(s) or question(s) the review addresses. | 2-3 |
| **METHODS** | | |  |
| Eligibility criteria | 5 | Specify the inclusion and exclusion criteria for the review and how studies were grouped for the syntheses. | 3 |
| Information sources | 6 | Specify all databases, registers, websites, organisations, reference lists and other sources searched or consulted to identify studies. Specify the date when each source was last searched or consulted. | 3 |
| Search strategy | 7 | Present the full search strategies for all databases, registers and websites, including any filters and limits used. | 3, tableS2 |
| Selection process | 8 | Specify the methods used to decide whether a study met the inclusion criteria of the review, including how many reviewers screened each record and each report retrieved, whether they worked independently, and if applicable, details of automation tools used in the process. | 3 |
| Data collection process | 9 | Specify the methods used to collect data from reports, including how many reviewers collected data from each report, whether they worked independently, any processes for obtaining or confirming data from study investigators, and if applicable, details of automation tools used in the process. | 3 |
| Data items | 10a | List and define all outcomes for which data were sought. Specify whether all results that were compatible with each outcome domain in each study were sought (e.g. for all measures, time points, analyses), and if not, the methods used to decide which results to collect. | 3 |
|  | 10b | List and define all other variables for which data were sought (e.g. participant and intervention characteristics, funding sources). Describe any assumptions made about any missing or unclear information. | 3 |
| Study risk of bias assessment | 11 | Specify the methods used to assess risk of bias in the included studies, including details of the tool(s) used, how many reviewers assessed each study and whether they worked independently, and if applicable, details of automation tools used in the process. | 4 |
| Effect measures | 12 | Specify for each outcome the effect measure(s) (e.g. risk ratio, mean difference) used in the synthesis or presentation of results. | 4 |
| Synthesis methods | 13a | Describe the processes used to decide which studies were eligible for each synthesis (e.g. tabulating the study intervention characteristics and comparing against the planned groups for each synthesis (item #5)). | 4 |
|  | 13b | Describe any methods required to prepare the data for presentation or synthesis, such as handling of missing summary statistics, or data conversions. | 4 |
|  | 13c | Describe any methods used to tabulate or visually display results of individual studies and syntheses. | 4 |
|  | 13d | Describe any methods used to synthesize results and provide a rationale for the choice(s). If meta-analysis was performed, describe the model(s), method(s) to identify the presence and extent of statistical heterogeneity, and software package(s) used. | 4 |
|  | 13e | Describe any methods used to explore possible causes of heterogeneity among study results (e.g. subgroup analysis, meta-regression). | 4 |
|  | 13f | Describe any sensitivity analyses conducted to assess robustness of the synthesized results. | 4 |
| Reporting bias assessment | 14 | Describe any methods used to assess risk of bias due to missing results in a synthesis (arising from reporting biases). | 4 |
| Certainty assessment | 15 | Describe any methods used to assess certainty (or confidence) in the body of evidence for an outcome. | 4 |
| **RESULTS** | | |  |
| Study selection | 16a | Describe the results of the search and selection process, from the number of records identified in the search to the number of studies included in the review, ideally using a flow diagram. | 4-5 |
|  | 16b | Cite studies that might appear to meet the inclusion criteria, but which were excluded, and explain why they were excluded. | 4-5 |
| Study characteristics | 17 | Cite each included study and present its characteristics. | 4-5 |
| Risk of bias in studies | 18 | Present assessments of risk of bias for each included study. | 5,11 |
| Results of individual studies | 19 | For all outcomes, present, for each study: (a) summary statistics for each group (where appropriate) and (b) an effect estimate and its precision (e.g. confidence/credible interval), ideally using structured tables or plots. | Table1-4 |
| Results of syntheses | 20a | For each synthesis, briefly summarise the characteristics and risk of bias among contributing studies. | 5-11 |
|  | 20b | Present results of all statistical syntheses conducted. If meta-analysis was done, present for each the summary estimate and its precision (e.g. confidence/credible interval) and measures of statistical heterogeneity. If comparing groups, describe the direction of the effect. | 5-11 |
|  | 20c | Present results of all investigations of possible causes of heterogeneity among study results. | 5-11 |
|  | 20d | Present results of all sensitivity analyses conducted to assess the robustness of the synthesized results. | 11 |
| Reporting biases | 21 | Present assessments of risk of bias due to missing results (arising from reporting biases) for each synthesis assessed. | 5-11 |
| Certainty of evidence | 22 | Present assessments of certainty (or confidence) in the body of evidence for each outcome assessed. | - |
| **DISCUSSION** | | |  |
| Discussion | 23a | Provide a general interpretation of the results in the context of other evidence. | 12-17 |
|  | 23b | Discuss any limitations of the evidence included in the review. | 17 |
|  | 23c | Discuss any limitations of the review processes used. | 17 |
|  | 23d | Discuss implications of the results for practice, policy, and future research. | 12-17 |
| **OTHER INFORMATION** | | |  |
| Registration and protocol | 24a | Provide registration information for the review, including register name and registration number, or state that the review was not registered. | 2 |
|  | 24b | Indicate where the review protocol can be accessed, or state that a protocol was not prepared. | - |
|  | 24c | Describe and explain any amendments to information provided at registration or in the protocol. | - |
| Support | 25 | Describe sources of financial or non-financial support for the review, and the role of the funders or sponsors in the review. | 18 |
| Competing interests | 26 | Declare any competing interests of review authors. | 18 |
| Availability of data, code and other materials | 27 | Report which of the following are publicly available and where they can be found: template data collection forms; data extracted from included studies; data used for all analyses; analytic code; any other materials used in the review. | 18 |

*From:*  Page MJ, McKenzie JE, Bossuyt PM, Boutron I, Hoffmann TC, Mulrow CD, et al. The PRISMA 2020 statement: an updated guideline for reporting systematic reviews. BMJ 2021;372:n71. doi: 10.1136/bmj.n71. This work is licensed under CC BY 4.0. To view a copy of this license, visit <https://creativecommons.org/licenses/by/4.0/>

**Supplementary Table 2.Search Strategy**

| Search Terms |
| --- |
| Based on PubMed, Embase, Web of Science databases, China National Knowledge Infrastructure, and Wanfang. |
| ((Pulmonary Cancer*[Title/Abstract])OR (Pulmonary Neoplasm*[Title/Abstract])OR (Lung  Neoplasm*[Title/Abstract])OR (Lung Cancer*[Title/Abstract])OR (Neoplasm of the lung[Title/Abstract])OR (Neoplasm of lung[Title/Abstract])OR (Cancer of Lung[Title/Abstract])OR (carcinoma of the lung[Title/Abstract])OR (lung carcinoma[Title/Abstract])OR (Cancer of the Lung[Title/Abstract]))  AND ((Immun*[Title/Abstract])OR (chemoimmun*[Title/Abstract])OR (PD[Title/Abstract])OR (pembrolizumab[Title/Abstract])OR (nivolumab[Title/Abstract])OR (atezolizumab[Title/Abstract]))  AND ((neoadjuvant[Title/Abstract])OR (preoperative therapy[Title/Abstract])OR (induction therapy[Title/Abstract])) |

**Supplementary Table 3. The table of Radiomics quality scores 2.0 for each study.**

| **Study ID** | **Criteria** | | **Scores** | **She** | **Liu** | **Han** | **Huang** | **Peng** | **Qu** | **Wang** | **Ye1** | **Ye2** | **Bao** | **Fan** | **Gan** | **Geng** | **Han** | **Xiong** | **Ye** | **Zheng** |
| --- | --- | --- | --- | --- | --- | --- | --- | --- | --- | --- | --- | --- | --- | --- | --- | --- | --- | --- | --- | --- |
| **Years** |  |  |  | **2022** | **2023** | **2024** | **2024** | **2024** | **2024** | **2024** | **2024** | **2024** | **2025** | **2025** | **2025** | **2025** | **2025** | **2025** | **2025** | **2025** |
| **Select Method** |  |  |  | Deep learning | Handcrafted Radiomics | Handcrafted Radiomics | Handcrafted Radiomics | Deep learning | Deep learning | Handcrafted Radiomics | Both | Handcrafted Radiomics | Handcrafted Radiomics | Handcrafted Radiomics | Both | Both | Handcrafted Radiomics | Handcrafted Radiomics | Both | Deep learning |
| RRL 1 – Foundational Exploration | 1 | Unmet Clinical Need | 2 | 2 | 2 | 2 | 2 | 2 | 2 | 2 | 2 | 2 | 2 | 2 | 2 | 2 | 2 | 2 | 2 | 2 |
|  | 2 | Hardware Description | 1 | 1 | 1 | 1 | 1 | 1 | 1 | 1 | 1 | 1 | 1 | 1 | 1 | 1 | 1 | 1 | 1 | 1 |
|  | 3 | Image Protocol Quality | 2 | 0 | 0 | 0 | 0 | 0 | 0 | 0 | 0 | 0 | 0 | 0 | 0 | 0 | 0 | 0 | 0 | 0 |
|  | 4 | Inclusion and Exclusion Criteria | 1 | 1 | 1 | 1 | 1 | 0 | 0 | 1 | 1 | 1 | 1 | 1 | 1 | 1 | 1 | 1 | 1 | 1 |
|  | 5 | Diversity and Distribution | 1 | 1 | 1 | 1 | 1 | 1 | 1 | 1 | 1 | 1 | 1 | 1 | 1 | 1 | 1 | 1 | 1 | 1 |
| RRL 2 – Data Preparation | 6 | Feature Robustness | 1 | N/A | 0 | 1 | 1 | N/A | N/A | 1 | 1 | 1 | 1 | 1 | 1 | 1 | 0 | 1 | 0 | N/A |
|  | 7 | Preprocessing of Images | 1 | 1 | 1 | 1 | 1 | 1 | 1 | 1 | 1 | 1 | 1 | 1 | 1 | 1 | 1 | 0 | 1 | 1 |
|  | 8 | Harmonization | 1 | 0 | 0 | 0 | 0 | 0 | 0 | 0 | 0 | 0 | 0 | 0 | 1 | 1 | 0 | 0 | 0 | 0 |
|  | 9 | Compliance with International Standards | 1 | N/A | 0 | 1 | 1 | N/A | N/A | 1 | 0 | 1 | 1 | 0 | 1 | 1 | 1 | 1 | 1 | N/A |
|  | 10 | Automatic Segmentation | 1 | 0 | 0 | 0 | 0 | 0 | 0 | 0 | 0 | 0 | 0 | 1 | 0 | 0 | 0 | 0 | 0 | 0 |
| RRL 3 – Prototype Model Development | 11 | Feature Reduction | 1 | N/A | 1 | 1 | 1 | N/A | N/A | 1 | 1 | 1 | 1 | 1 | 1 | 1 | 1 | 1 | 1 | N/A |
|  | 12 | Feature Robustness for Feature Selection | 1 | N/A | 1 | 1 | 1 | N/A | N/A | 1 | 0 | 1 | 1 | 0 | 0 | 0 | 0 | 1 | 1 | N/A |
|  | 13 | HCR + DL Combination | 1 | 0 | 0 | 0 | 0 | 1 | 0 | 0 | 0 | 0 | 0 | 0 | 1 | 1 | 0 | 0 | 1 | 0 |
|  | 14 | Multivariable Analysis | 2 | 2 | 2 | 2 | 2 | 2 | 0 | 2 | 0 | 0 | 0 | 2 | 0 | 0 | 2 | 2 | 2 | 2 |
| RRL 4 – Internal Validation | 15 | Single Center Validation | 1 | 1 | 1 | 1 | 1 | 0 | 1 | 1 | 1 | 0 | 1 | 1 | 1 | 1 | 1 | 1 | 1 | 0 |
|  | 16 | Cut-off Analyses | 1 | 1 | 0 | 0 | 0 | 1 | 0 | 1 | 1 | 0 | 1 | 1 | 0 | 0 | 1 | 1 | 1 | 1 |
|  | 17 | Discrimination Statistics | 2 | 2 | 2 | 2 | 2 | 2 | 2 | 2 | 2 | 2 | 2 | 2 | 2 | 1 | 2 | 2 | 2 | 2 |
|  | 18 | Calibration Statistics | 1 | 0 | 1 | 1 | 1 | 0 | 1 | 1 | 0 | 1 | 1 | 0 | 0 | 1 | 1 | 1 | 1 | 0 |
|  | 19 | Failure Mode Analysis | 1 | 1 | 1 | 1 | 1 | 1 | 1 | 1 | 1 | 1 | 1 | 1 | 1 | 1 | 1 | 1 | 1 | 1 |
|  | 20 | Open Science and Data | 3 | 1 | 0 | 0 | 0 | 0 | 0 | 0 | 1 | 1 | 0 | 0 | 0 | 0 | 0 | 0 | 0 | 0 |
| RRL 5 – Capability Testing | 21 | Multi‑center Validation | 3 | 2 | 0 | 3 | 2 | 2 | 2 | 2 | 2 | 2 | 2 | 0 | 2 | 2 | 2 | 0 | 2 | 2 |
|  | 22 | Comparison with ‘Current Clinical Standard’ | 2 | 2 | 2 | 2 | 2 | 2 | 2 | 2 | 2 | 2 | 2 | 2 | 2 | 2 | 2 | 2 | 2 | 2 |
|  | 23 | Comparison to Previous Work | 1 | 1 | 1 | 0 | 1 | 1 | 1 | 1 | 1 | 1 | 1 | 1 | 1 | 1 | 1 | 1 | 1 | 1 |
|  | 24 | Potential Clinical Utility | 2 | 0 | 2 | 2 | 2 | 0 | 2 | 2 | 0 | 2 | 2 | 0 | 2 | 2 | 2 | 2 | 2 | 0 |
| RRL 6 – Trustworthiness Assessment | 25 | Explainability | 1 | 1 | 1 | 1 | 1 | 1 | 0 | 1 | 1 | 0 | 0 | 0 | 1 | 1 | 0 | 0 | 1 | 0 |
|  | 26 | Explainability Evaluation | 1 | 0 | 0 | 0 | 0 | 0 | 0 | 0 | 0 | 0 | 0 | 0 | 0 | 1 | 0 | 0 | 1 | 0 |
|  | 27 | Biological Correlates | 1 | 1 | 1 | 1 | 1 | 1 | 1 | 1 | 1 | 0 | 1 | 1 | 1 | 1 | 0 | 1 | 1 | 0 |
|  | 28 | Fairness Evaluation and Mitigation | 2 | 0 | 0 | 0 | 0 | 0 | 0 | 0 | 0 | 0 | 0 | 0 | 0 | 0 | 0 | 0 | 0 | 0 |
| RRL 7 – Prospective Validity | 29 | Usability for Clinicians | 1 | 0 | 0 | 0 | 0 | 0 | 0 | 1 | 0 | 0 | 0 | 0 | 0 | 0 | 0 | 0 | 1 | 1 |
|  | 30 | Sample Size Calculation | 1 | 0 | 0 | 0 | 0 | 0 | 0 | 0 | 0 | 0 | 0 | 0 | 0 | 0 | 0 | 0 | 0 | 0 |
|  | 31 | Clinical Trial Pre-registration | 1 | 0 | 0 | 0 | 0 | 0 | 0 | 0 | 0 | 1 | 0 | 0 | 0 | 0 | 0 | 0 | 1 | 0 |
|  | 32 | Prospective Validation | 3 | 0 | 0 | 0 | 0 | 0 | 0 | 0 | 0 | 0 | 0 | 0 | 0 | 0 | 0 | 0 | 0 | 3 |
|  | 33 | Real‑World Clinical Assessment | 1 | 0 | 0 | 0 | 0 | 0 | 0 | 0 | 0 | 0 | 0 | 0 | 0 | 0 | 0 | 0 | 0 | 1 |
| RRL 8 – Applicability and Sustainability | 34 | Software Traceability | 1 | 0 | 0 | 0 | 0 | 0 | 0 | 1 | 0 | 0 | 0 | 0 | 0 | 0 | 0 | 0 | 0 | 0 |
|  | 35 | Software Safeguards | 1 | 0 | 0 | 0 | 0 | 0 | 0 | 0 | 0 | 0 | 0 | 0 | 0 | 0 | 0 | 0 | 0 | 0 |
|  | 36 | Cost‑effectiveness Analysis | 2 | 0 | 0 | 0 | 0 | 0 | 0 | 0 | 0 | 0 | 0 | 0 | 0 | 0 | 0 | 0 | 0 | 0 |
|  | 37 | Performance Drift | 1 | 0 | 0 | 0 | 0 | 0 | 0 | 0 | 0 | 0 | 0 | 0 | 0 | 0 | 0 | 0 | 0 | 0 |
|  | 38 | Continuous Learning | 1 | 0 | 0 | 0 | 0 | 0 | 0 | 0 | 0 | 0 | 0 | 0 | 0 | 0 | 0 | 0 | 0 | 0 |
| RRL 9 – Clinical Deployment | 39 | Define the Level of Automation in Clinical Practice | 1 | 1 | 0 | 0 | 0 | 0 | 0 | 0 | 0 | 0 | 1 | 0 | 0 | 0 | 0 | 0 | 0 | 1 |
|  | 40 | Quality Management System | 1 | 0 | 0 | 0 | 0 | 0 | 0 | 0 | 0 | 0 | 0 | 0 | 0 | 0 | 0 | 0 | 0 | 0 |
|  | 41 | Regulatory Requirements | 1 | 0 | 0 | 0 | 0 | 0 | 0 | 0 | 0 | 0 | 0 | 0 | 0 | 0 | 0 | 0 | 0 | 0 |
|  | 42 | Product on the Market | 1 | 0 | 0 | 0 | 0 | 0 | 0 | 0 | 0 | 0 | 0 | 0 | 0 | 0 | 0 | 0 | 0 | 0 |
|  | Total | | 56 | 22 | 22 | 26 | 26 | 19 | 18 | 29 | 21 | 23 | 25 | 20 | 24 | 25 | 23 | 23 | 30 | 23 |

**Supplementary Table 4. PRISMA checklist of this Meta-analysis.**

Title: CT-Based Radiomics in Predicting the Efficacy of Preoperative Neoadjuvant chemoimmunotherapy in Non-Small Cell Lung Cancer: A Systematic Review and Meta-Analysis

Question: Lung cancer is one of the most commonly diagnosed cancers and the

leading cause of cancer-related deaths globally. Recently, neoadjuvant chemoimmunotherapy has emerged as a significant research direction in solid tumor treatment, achieving notable progress in the clinical management of non-small cell lung cancer (NSCLC). Clinical trials have established that major pathological response (MPR) and pathological complete response (pCR) serve as key surrogate endpoints for evaluating chemoimmunotherapy efficacy. However, a substantial proportion of patients fail to benefit from neoadjuvant chemoimmunotherapy. Consequently, there is an urgent need for an alternative method to predict response to neoadjuvant treatment. Furthermore, accurate prediction of neoadjuvant chemoimmunotherapy outcomes enables timely formulation of appropriate treatment strategies. This avoids potential undertreatment or overtreatment and prevents unnecessary complications associated with surgery. Unfortunately, reliable biomarkers for predicting pathological response after neoadjuvant therapy in resectable NSCLC are currently unavailable. CT, as a non-invasive imaging modality, has been widely adopted for preoperative evaluation of NSCLC and has demonstrated applicability in assessing pathological response to NSCLC treatment. Artificial intelligence has shown promising performance in predicting treatment efficacy. However, the lack of standardized radiomics workflows limits the robustness and reproducibility of these models.

This study aims to systematically review and comprehensively summarize the application of CT in predicting the efficacy of preoperative neoadjuvant chemoimmunotherapy for NSCLC, focusing on its diagnostic performance, sensitivity, and specificity. It seeks to provide clinicians with a potential reference tool for evaluating the efficacy of neoadjuvant chemoimmunotherapy, thereby facilitating the development of personalized treatment strategies.

Keywords:Non-Small Cell Lung Cancer; radiomics; neoadjuvant; chemoimmunotherapy; Meta-Analysis

**18. * Condition or domain being studied.**

Detecting clinical efficacy following neoadjuvant immunochemotherapy for non-small cell lung cancer.

**19. * Participants/population.**

People who were diagnosed with NSCLC.

**20. * Intervention(s), exposure(s).**

Undergo CT scanning prior to neoadjuvant therapy, receive at least one cycle of immunotherapy combined with chemotherapy, and perform surgical treatment with a complete pathology report preserved.

**21. * Comparator(s)/control.**

Histopathologic results were used as the reference standard to compare the performance of radiomics models.

**22. * Types of study to be included**

Only nonrandomized study types will be included.

Included Observational studies

Excluded non-English publications, case reports, editorials, conference abstracts, review articles

**Context:** The new adjuvant immunochemotherapy regimen does not impose uniform requirements on immunotherapy and chemotherapy regimens.

**24. * Main outcome(s).**

The main outcome measures encompassed sensitivity, specificity, positive likelihood ratio, negative likelihood ratio, and the area under the curve

Measures of effect

sensitivity, specificity, accuracy, and the area under the receiver operating characteristic curve (AUC).

1. *** Additional outcome(s).**

NA

**26. * Data extraction (selection and coding).**

Two independent reviewers extracted data from the included cohort study. The extracted information included: (1) general data: authors’ names, year of publication, country, and sample size; (2) participant characteristics: age, sex, diagnosis, and neoadjuvant chemoimmunotherapy regimen(if any); (3) intervention and control group details:imaging modality; and (4) outcomes: diagnostic performance metrics (Accuracy, sensitivity, specificity, AUC). Any discrepancies in data extraction were resolved through discussion and consensus between the reviewers.

**27. * Risk of bias (quality) assessment.**

Use the Radiomics Quality Score (RQS) checklist and the modified Quality Assessment of Diagnostic Accuracy Studies (QUADAS-2) tool to assess the included articles.

**28. * Strategy for data synthesis.**

1.Statistical analysis was performed using STATA 14.0 (StataCorp LLC, College Station, Texas, USA). A summary receiver operating characteristic (ROC) curve was created by analyzing 2 × 2 table data, with AUC as the measure of diagnostic accuracy.

2.The inconsistency index (*I²*) statistic, a kind of index to represent the percent of diversity that is due to heterogeneity rather than chance, was applied to quantify the magnitude of heterogeneity derived from the random-effects Mantel-Haenszel model. It indicated the significant heterogeneity if the *I²* is greater than 50%.

3.Deek funnel plots were used to examine potential publication bias, with the Egger test quantitatively assessing the risk of such bias. Additionally, we applied Fagan plots to evaluate clinical utility, providing prior probabilities for predicting neoadjuvant efficacy when calculating posterior probabilities. Statistical significance was defined as P < 0.05.

4.A stepwise sensitivity analysis was conducted by sequentially omitting one study at a time to assess the impact of individual studies on the overall estimate.

We will assess the certainty of the results using tools such as heterogeneity analysis, sensitivity analysis, publication bias assessment, and the QUDAS scale.

**29. * Analysis of subgroups or subsets.**

In subgroup analyses, the source of heterogeneity is determined by examining specific covariates. Evaluated factors include: study setting (multicenter or single-center), presence of external validation cohorts, localization methods, segmentation techniques and dimensions, classifier types employed, whether images underwent normalized processing, type of predicted outcome metric (pCR or MRP), and whether prediction models incorporated clinical factors.

Identifier: CRD420251174128

**Supplementary Table 5.** **The fundamental characteristics incorporated into the validation model.**

| Author | Year | Country | Tp | Fp | Fn | Tn | Sensitivity | Specificity | AUC | Data source | External validation | AI method | Imaging equipment | Study design | Normalized | Combined clinic | ICC | RQS | ROI |
| --- | --- | --- | --- | --- | --- | --- | --- | --- | --- | --- | --- | --- | --- | --- | --- | --- | --- | --- | --- |
| She(ex)(com) | 2022 | China | 42 | 9 | 6 | 14 | 0.87 | 0.61 | 0.75 | multi-center | Yes | DL | CT | MPR | Yes | Yes | No | 16 | 3D |
| She(in)(com) | 2022 | China | 16 | 2 | 14 | 29 | 0.53 | 0.94 | 0.77 | single-center | No | DL | CT | MPR | Yes | Yes | No | 16 | 3D |
| She(ex)(DL) | 2022 | China | 44 | 10 | 4 | 13 | 0.92 | 0.57 | 0.72 | multi-center | Yes | DL | CT | MPR | Yes | No | No | 16 | 3D |
| She(in)(DL) | 2022 | China | 21 | 7 | 9 | 24 | 0.70 | 0.78 | 0.73 | single-center | No | DL | CT | MPR | Yes | No | No | 16 | 3D |
| She(ex)(clinical) | 2022 | China | 21 | 3 | 27 | 20 | 0.44 | 0.88 | 0.65 | multi-center | Yes | DL | CT | MPR | Yes | Yes | No | 16 | 3D |
| She(in)(clinical) | 2022 | China | 23 | 12 | 7 | 19 | 0.76 | 0.61 | 0.63 | single-center | No | DL | CT | MPR | Yes | Yes | No | 16 | 3D |
| Liu(in)(com) | 2023 | China | 12 | 1 | 4 | 8 | 0.75 | 0.89 | 0.81 | single-center | No | ML | CT | MPR | No | Yes | Yes | 16 | 3D |
| Liu(in)(radiomics) | 2024 | China | 13 | 2 | 3 | 7 | 0.81 | 0.78 | 0.76 | single-center | No | ML | CT | MPR | No | No | Yes | 16 | 3D |
| Liu(in)(clinical) | 2025 | China | 14 | 5 | 2 | 4 | 0.88 | 0.44 | 0.66 | single-center | No | ML | CT | MPR | No | Yes | Yes | 16 | 3D |
| Han(ex2)(com) | 2024 | China | 6 | 2 | 1 | 12 | 0.80 | 0.83 | 0.85 | single-center | Yes | ML | contrast-enhanced | MPR | Yes | Yes | Yes | 19 | 3D |
| Han(ex3)(com) | 2024 | China | 10 | 7 | 0 | 4 | 1.00 | 0.36 | 0.67 | single-center | Yes | ML | contrast-enhanced | MPR | Yes | Yes | Yes | 19 | 3D |
| Han(in1)(com) | 2024 | China | 26 | 12 | 0 | 12 | 1.00 | 0.50 | 0.76 | single-center | No | ML | contrast-enhanced | MPR | Yes | Yes | Yes | 19 | 3D |
| Han(ex2)(pre) | 2024 | China | 6 | 6 | 1 | 8 | 0.80 | 0.60 | 0.48 | single-center | Yes | ML | contrast-enhanced | MPR | Yes | Yes | Yes | 19 | 3D |
| Han(ex3)(pre) | 2024 | China | 4 | 1 | 6 | 10 | 0.38 | 0.91 | 0.61 | single-center | Yes | ML | contrast-enhanced | MPR | Yes | Yes | Yes | 19 | 3D |
| Han(in1)(pre) | 2024 | China | 10 | 3 | 16 | 21 | 0.39 | 0.88 | 0.62 | single-center | No | ML | contrast-enhanced | MPR | Yes | Yes | Yes | 19 | 3D |
| Han(ex2)(iRECIST) | 2024 | China | 6 | 7 | 1 | 7 | 0.80 | 0.50 | 0.65 | single-center | Yes | ML | contrast-enhanced | MPR | Yes | No | Yes | 19 | 3D |
| Han(ex3)(iRECIST) | 2024 | China | 0 | 0 | 10 | 11 | 0.00 | 1.00 | 0.47 | single-center | Yes | ML | contrast-enhanced | MPR | Yes | No | Yes | 19 | 3D |
| Han(in1)(iRECIST) | 2024 | China | 20 | 15 | 6 | 9 | 0.77 | 0.38 | 0.57 | single-center | No | ML | contrast-enhanced | MPR | Yes | No | Yes | 19 | 3D |
| Han(ex2)(deta-radiomics) | 2024 | China | 6 | 2 | 1 | 12 | 0.80 | 0.83 | 0.83 | single-center | Yes | ML | contrast-enhanced | MPR | Yes | No | Yes | 19 | 3D |
| Han(ex3)(deta-radiomics) | 2024 | China | 6 | 2 | 4 | 9 | 0.63 | 0.82 | 0.72 | single-center | Yes | ML | contrast-enhanced | MPR | Yes | No | Yes | 19 | 3D |
| Han(in1)(deta-radiomics) | 2024 | China | 22 | 11 | 4 | 13 | 0.85 | 0.54 | 0.73 | single-center | No | ML | contrast-enhanced | MPR | Yes | No | Yes | 19 | 3D |
| Huang(ex)(CRC） | 2024 | China | 15 | 3 | 7 | 18 | 0.68 | 0.86 | 0.77 | single-center | Yes | ML | CT | MPR | Yes | Yes | Yes | 17 | 2D |
| Huang(in)(T） | 2024 | China | 72 | 22 | 4 | 7 | 0.95 | 0.25 | 0.56 | single-center | No | ML | CT | MPR | Yes | No | Yes | 17 | 2D |
| Huang(in)(clinical） | 2024 | China | 53 | 12 | 23 | 17 | 0.70 | 0.59 | 0.62 | single-center | No | ML | CT | MPR | Yes | Yes | Yes | 17 | 2D |
| Huang(in)(P1） | 2024 | China | 72 | 23 | 4 | 6 | 0.95 | 0.20 | 0.71 | single-center | No | ML | CT | MPR | Yes | No | Yes | 17 | 2D |
| Huang(in)(P2） | 2024 | China | 51 | 10 | 25 | 19 | 0.67 | 0.66 | 0.66 | single-center | No | ML | CT | MPR | Yes | No | Yes | 17 | 2D |
| Huang(in)(P3） | 2024 | China | 71 | 21 | 5 | 8 | 0.93 | 0.27 | 0.74 | single-center | No | ML | CT | MPR | Yes | No | Yes | 17 | 2D |
| Huang(in)(CR） | 2024 | China | 70 | 14 | 6 | 15 | 0.92 | 0.53 | 0.81 | single-center | No | ML | CT | MPR | Yes | No | Yes | 17 | 2D |
| Huang(in)(CRC） | 2024 | China | 72 | 13 | 4 | 16 | 0.95 | 0.57 | 0.81 | single-center | No | ML | CT | MPR | Yes | Yes | Yes | 17 | 2D |
| Peng(ex1) | 2024 | China | 28 | 0 | 4 | 28 | 0.88 | 1.00 | 0.95 | multi-center | Yes | DL | contrast-enhanced | MPR | Yes | No | No | 14 | 2D |
| Peng(ex2) | 2025 | China | 18 | 3 | 3 | 25 | 0.86 | 0.90 | 0.90 | multi-center | Yes | DL | contrast-enhanced | MPR | Yes | No | No | 14 | 2D |
| Qu(ex)(DL) | 2024 | China | 19 | 22 | 5 | 29 | 0.79 | 0.57 | 0.74 | multi-center | Yes | DL | CT | pCR | Yes | No | No | 18 | 3D |
| Qu(ex)(clinical) | 2024 | China | 22 | 31 | 2 | 20 | 0.92 | 0.39 | 0.57 | multi-center | Yes | ML | CT | pCR | Yes | Yes | No | 18 | 3D |
| Qu(in)(DL) | 2024 | China | 18 | 12 | 6 | 33 | 0.75 | 0.73 | 0.78 | single-center | No | DL | CT | pCR | Yes | No | No | 18 | 3D |
| Qu(in)(clinical) | 2024 | China | 1 | 5 | 23 | 40 | 0.04 | 0.89 | 0.58 | single-center | No | ML | CT | pCR | Yes | Yes | No | 18 | 3D |
| Wang(in)(com) | 2024 | China | 31 | 8 | 6 | 18 | 0.84 | 0.69 | 0.80 | single-center | No | ML | CT | MPR | Yes | Yes | Yes | 18 | 3D |
| Wang(in)(rad) | 2024 | China | 28 | 15 | 9 | 11 | 0.76 | 0.42 | 0.60 | single-center | No | ML | CT | MPR | Yes | No | Yes | 18 | 3D |
| Wang(in)(clinical) | 2024 | China | 31 | 7 | 6 | 19 | 0.84 | 0.73 | 0.80 | single-center | No | ML | CT | MPR | Yes | Yes | Yes | 18 | 3D |
| Ye1(ex)(fCT) | 2024 | China | 34 | 20 | 3 | 55 | 0.92 | 0.74 | 0.87 | multi-center | Yes | DL | CT | pCR | Yes | No | Yes | 15 | 2D |
| Ye1(in)(fCT) | 2024 | China | 37 | 27 | 0 | 48 | 1.00 | 0.64 | 0.88 | single-center | No | DL | CT | pCR | Yes | No | Yes | 15 | 2D |
| Ye1(ex)(uCT) | 2024 | China | 35 | 35 | 2 | 40 | 0.96 | 0.53 | 0.76 | multi-center | Yes | DL | CT | pCR | Yes | No | Yes | 15 | 2D |
| Ye1(in)(uCT) | 2024 | China | 37 | 21 | 0 | 54 | 1.00 | 0.71 | 0.83 | single-center | No | DL | CT | pCR | Yes | No | Yes | 15 | 2D |
| Ye1(ex)(eCT) | 2024 | China | 31 | 26 | 6 | 50 | 0.83 | 0.66 | 0.80 | multi-center | Yes | DL | contrast-enhanced | pCR | Yes | No | Yes | 15 | 2D |
| Ye1(in)(eCT) | 2024 | China | 32 | 16 | 5 | 59 | 0.88 | 0.79 | 0.84 | single-center | No | DL | contrast-enhanced | pCR | Yes | No | Yes | 15 | 2D |
| Ye2(ex)(habitat) | 2024 | China | 18 | 13 | 5 | 34 | 0.78 | 0.72 | 0.78 | multi-center | Yes | ML | contrast-enhanced | pCR | Yes | No | Yes | 16 | 3D |
| Ye2(ex)(rad) | 2025 | China | 18 | 16 | 6 | 32 | 0.74 | 0.66 | 0.72 | multi-center | Yes | ML | contrast-enhanced | pCR | Yes | No | Yes | 16 | 3D |
| Bao(ex)(com) | 2025 | China | 31 | 4 | 25 | 26 | 0.55 | 0.87 | 0.75 | single-center | Yes | ML | contrast-enhanced | MPR | Yes | No | No | 16 | 3D |
| Bao(ex)(deta-rad) | 2025 | China | 34 | 8 | 22 | 22 | 0.61 | 0.73 | 0.72 | single-center | Yes | ML | contrast-enhanced | MPR | Yes | No | No | 16 | 3D |
| Bao(ex)(pre) | 2025 | China | 21 | 4 | 35 | 26 | 0.38 | 0.87 | 0.64 | single-center | Yes | ML | contrast-enhanced | MPR | Yes | No | No | 16 | 3D |
| Bao(ex)(post) | 2025 | China | 37 | 9 | 19 | 21 | 0.66 | 0.70 | 0.69 | single-center | Yes | ML | contrast-enhanced | MPR | Yes | No | No | 16 | 3D |
| Bao(ex)(RECIST) | 2025 | China | 12 | 4 | 44 | 26 | 0.21 | 0.87 | 0.55 | single-center | Yes | ML | contrast-enhanced | MPR | Yes | No | No | 16 | 3D |
| Bao(in)(com) | 2025 | China | 31 | 11 | 11 | 27 | 0.74 | 0.71 | 0.77 | single-center | No | ML | contrast-enhanced | MPR | Yes | No | No | 16 | 3D |
| Bao(in)(deta-rad) | 2025 | China | 30 | 12 | 12 | 26 | 0.71 | 0.68 | 0.74 | single-center | No | ML | contrast-enhanced | MPR | Yes | No | No | 16 | 3D |
| Bao(in)(pre) | 2025 | China | 23 | 14 | 19 | 24 | 0.55 | 0.63 | 0.63 | single-center | No | ML | contrast-enhanced | MPR | Yes | No | No | 16 | 3D |
| Bao(in)(post) | 2025 | China | 30 | 18 | 12 | 20 | 0.71 | 0.53 | 0.68 | single-center | No | ML | contrast-enhanced | MPR | Yes | No | No | 16 | 3D |
| Bao(in)(RECIST) | 2025 | China | 22 | 9 | 20 | 29 | 0.52 | 0.76 | 0.68 | single-center | No | ML | contrast-enhanced | MPR | Yes | No | No | 16 | 3D |
| Fan(in)(T) | 2025 | China | 27 | 17 | 6 | 15 | 0.82 | 0.48 | 0.73 | single-center | No | ML | CT | pCR | Yes | No | No | 12 | 2D |
| Fan(in)(P3) | 2025 | China | 24 | 13 | 9 | 19 | 0.73 | 0.59 | 0.71 | single-center | No | ML | CT | pCR | Yes | No | No | 12 | 2D |
| Fan(in)(P6) | 2025 | China | 22 | 13 | 11 | 19 | 0.67 | 0.59 | 0.66 | single-center | No | ML | CT | pCR | Yes | No | No | 12 | 2D |
| Fan(in)(T+P3) | 2025 | China | 19 | 7 | 14 | 25 | 0.58 | 0.77 | 0.76 | single-center | No | ML | CT | pCR | Yes | No | No | 12 | 2D |
| Fan(in)(T+P6) | 2025 | China | 19 | 16 | 14 | 16 | 0.58 | 0.50 | 0.68 | single-center | No | ML | CT | pCR | Yes | No | No | 12 | 2D |
| Gan(in)(rad) | 2025 | China | 16 | 3 | 1 | 15 | 0.92 | 0.82 | 0.91 | single-center | No | DL | contrast-enhanced | MPR | Yes | No | Yes |  | 2D and 3D |
| Gan(ex)(rad) | 2025 | China | 53 | 31 | 6 | 63 | 0.90 | 0.67 | 0.84 | multi-center | Yes | DL | contrast-enhanced | MPR | Yes | No | Yes |  | 2D and 3D |
| Gan(in)(ViT) | 2025 | China | 13 | 2 | 4 | 16 | 0.77 | 0.86 | 0.89 | single-center | No | DL | contrast-enhanced | MPR | Yes | No | Yes |  | 2D and 3D |
| Gan(ex)(ViT) | 2025 | China | 42 | 20 | 17 | 74 | 0.71 | 0.79 | 0.79 | multi-center | Yes | DL | contrast-enhanced | MPR | Yes | No | Yes |  | 2D and 3D |
| Gan(in)(Tempo) | 2025 | China | 12 | 1 | 5 | 17 | 0.69 | 0.94 | 0.93 | single-center | No | DL | contrast-enhanced | MPR | Yes | No | Yes |  | 2D and 3D |
| Gan(ex)(Tempo) | 2025 | China | 52 | 23 | 7 | 71 | 0.88 | 0.76 | 0.85 | multi-center | Yes | DL | contrast-enhanced | MPR | Yes | No | Yes |  | 2D and 3D |
| Gan(in)(patho) | 2025 | China | 12 | 2 | 5 | 16 | 0.69 | 0.86 | 0.83 | single-center | No | DL | contrast-enhanced | MPR | Yes | No | Yes |  | 2D and 3D |
| Gan(ex)(patho) | 2025 | China | 52 | 41 | 7 | 53 | 0.88 | 0.56 | 0.75 | multi-center | Yes | DL | contrast-enhanced | MPR | Yes | No | Yes |  | 2D and 3D |
| Gan(in)(trans) | 2025 | China | 13 | 2 | 4 | 16 | 0.77 | 0.86 | 0.92 | single-center | No | DL | contrast-enhanced | MPR | Yes | No | Yes |  | 2D and 3D |
| Gan(ex)(trans) | 2025 | China | 53 | 29 | 6 | 65 | 0.90 | 0.69 | 0.86 | multi-center | Yes | DL | contrast-enhanced | MPR | Yes | No | Yes |  | 2D and 3D |
| Geng(ex)(Fusion) | 2025 | China | 59 | 8 | 1 | 48 | 0.98 | 0.86 | 0.92 | multi-center | Yes | DL | CT | MPR | Yes | No | Yes | 16 | 3D |
| Geng(ex)(MPI) | 2025 | China | 52 | 8 | 8 | 48 | 0.87 | 0.86 | 0.86 | multi-center | Yes | DL | CT | MPR | Yes | No | Yes | 16 | 3D |
| Geng(ex)(MSI) | 2025 | China | 46 | 7 | 14 | 49 | 0.77 | 0.88 | 0.84 | multi-center | Yes | DL | CT | MPR | Yes | No | Yes | 16 | 3D |
| Geng(ex)(MCI) | 2025 | China | 49 | 12 | 11 | 44 | 0.82 | 0.79 | 0.81 | multi-center | Yes | DL | CT | MPR | Yes | No | Yes | 16 | 3D |
| Geng(ex)(MAI) | 2025 | China | 49 | 15 | 11 | 41 | 0.82 | 0.73 | 0.79 | multi-center | Yes | DL | CT | MPR | Yes | No | Yes | 16 | 3D |
| Geng(ex)(ET) | 2025 | China | 43 | 18 | 17 | 38 | 0.72 | 0.68 | 0.74 | multi-center | Yes | ML | CT | MPR | Yes | No | Yes | 16 | 3D |
| Geng(ex)(SVM) | 2025 | China | 28 | 2 | 32 | 54 | 0.47 | 0.96 | 0.73 | multi-center | Yes | ML | CT | MPR | Yes | No | Yes | 16 | 3D |
| Geng(in)(Fusion) | 2025 | China | 22 | 2 | 2 | 18 | 0.92 | 0.90 | 0.97 | single-center | No | DL | CT | MPR | Yes | No | Yes | 16 | 3D |
| Geng(in)(MPI) | 2025 | China | 20 | 0 | 4 | 20 | 0.83 | 1.00 | 0.91 | single-center | No | DL | CT | MPR | Yes | No | Yes | 16 | 3D |
| Geng(in)(MSI) | 2025 | China | 20 | 3 | 4 | 17 | 0.83 | 0.85 | 0.86 | single-center | No | DL | CT | MPR | Yes | No | Yes | 16 | 3D |
| Geng(in)(MCI) | 2025 | China | 20 | 3 | 4 | 17 | 0.83 | 0.85 | 0.85 | single-center | No | DL | CT | MPR | Yes | No | Yes | 16 | 3D |
| Geng(in)(MAI) | 2025 | China | 19 | 3 | 5 | 17 | 0.79 | 0.85 | 0.87 | single-center | No | DL | CT | MPR | Yes | No | Yes | 16 | 3D |
| Han(in)(clinical) | 2025 | China | 37 | 15 | 6 | 3 | 0.86 | 0.17 | 0.55 | single-center | No | ML | contrast-enhanced | MPR | Yes | Yes | No | 24 | 3D |
| Han(in)(P6) | 2025 | China | 42 | 15 | 1 | 3 | 0.98 | 0.17 | 0.55 | single-center | No | ML | contrast-enhanced | MPR | Yes | No | No | 24 | 3D |
| Han(in)(habitat) | 2025 | China | 30 | 5 | 13 | 13 | 0.70 | 0.72 | 0.82 | single-center | No | ML | contrast-enhanced | MPR | Yes | No | No | 24 | 3D |
| Han(in)(N) | 2025 | China | 39 | 9 | 4 | 9 | 0.91 | 0.50 | 0.83 | single-center | No | ML | contrast-enhanced | MPR | Yes | Yes | No | 24 | 3D |
| Han(ex)(clinical) | 2025 | China | 20 | 12 | 3 | 1 | 0.87 | 0.08 | 0.62 | multi-center | Yes | ML | contrast-enhanced | MPR | Yes | Yes | No | 24 | 3D |
| Han(ex)(P6) | 2025 | China | 18 | 10 | 5 | 3 | 0.78 | 0.23 | 0.61 | multi-center | Yes | ML | contrast-enhanced | MPR | Yes | No | No | 24 | 3D |
| Han(ex)(habitat) | 2025 | China | 14 | 4 | 9 | 9 | 0.61 | 0.69 | 0.77 | multi-center | Yes | ML | contrast-enhanced | MPR | Yes | No | No | 24 | 3D |
| Han(ex)(N) | 2025 | China | 21 | 6 | 2 | 7 | 0.91 | 0.54 | 0.80 | multi-center | Yes | ML | contrast-enhanced | MPR | Yes | Yes | No | 24 | 3D |
| Xiong(in)(com) | 2025 | China | 30 | 19 | 13 | 53 | 0.70 | 0.74 | 0.74 | single-center | No | ML | contrast-enhanced | pCR | No | Yes | Yes | 17 | 3D |
| Xiong(in)(Rad) | 2025 | China | 30 | 19 | 13 | 53 | 0.70 | 0.74 | 0.72 | single-center | No | ML | contrast-enhanced | pCR | No | No | Yes | 17 | 3D |
| Ye(in)(pre-rad) | 2025 | China | 24 | 19 | 6 | 29 | 0.80 | 0.60 | 0.72 | multi-center | No | DL | contrast-enhanced | pCR | Yes | No | No |  | 3D |
| Ye(in)(pre-dl) | 2025 | China | 17 | 12 | 13 | 36 | 0.57 | 0.75 | 0.65 | multi-center | No | DL | contrast-enhanced | pCR | Yes | No | No |  | 3D |
| Ye(in)(pre-hi) | 2025 | China | 27 | 17 | 3 | 31 | 0.90 | 0.64 | 0.81 | multi-center | No | DL | contrast-enhanced | pCR | Yes | No | No |  | 3D |
| Ye(in)(post-rad) | 2025 | China | 12 | 6 | 18 | 42 | 0.40 | 0.88 | 0.64 | multi-center | No | DL | contrast-enhanced | pCR | Yes | No | No |  | 3D |
| Ye(in)(post-dl) | 2025 | China | 23 | 33 | 7 | 15 | 0.77 | 0.31 | 0.48 | multi-center | No | DL | contrast-enhanced | pCR | Yes | No | No |  | 3D |
| Ye(in)(post-hi) | 2025 | China | 28 | 16 | 2 | 32 | 0.92 | 0.67 | 0.85 | multi-center | No | DL | contrast-enhanced | pCR | Yes | No | No |  | 3D |
| Ye(in)(delta-rad) | 2025 | China | 19 | 6 | 11 | 42 | 0.63 | 0.87 | 0.81 | multi-center | No | DL | contrast-enhanced | pCR | Yes | No | No |  | 3D |
| Ye(in)(delta-dl) | 2025 | China | 13 | 3 | 17 | 45 | 0.43 | 0.94 | 0.74 | multi-center | No | DL | contrast-enhanced | pCR | Yes | No | No |  | 3D |
| Ye(in)(delta-hi) | 2025 | China | 25 | 26 | 5 | 22 | 0.83 | 0.46 | 0.64 | multi-center | No | DL | contrast-enhanced | pCR | Yes | No | No |  | 3D |
| Ye(in)(nicerα) | 2025 | China | 18 | 9 | 12 | 39 | 0.60 | 0.81 | 0.72 | multi-center | No | DL | contrast-enhanced | pCR | Yes | No | No |  | 3D |
| Ye(in)(post) | 2025 | China | 24 | 18 | 6 | 30 | 0.80 | 0.62 | 0.76 | multi-center | No | DL | contrast-enhanced | pCR | Yes | No | No |  | 3D |
| Ye(in)(delta) | 2025 | China | 18 | 2 | 12 | 46 | 0.60 | 0.96 | 0.83 | multi-center | No | DL | contrast-enhanced | pCR | Yes | No | No |  | 3D |
| Ye(in)(nicerδ) | 2025 | China | 27 | 11 | 3 | 37 | 0.90 | 0.77 | 0.89 | multi-center | No | DL | contrast-enhanced | pCR | Yes | No | No |  | 3D |
| Ye(ex)(pre-rad) | 2025 | China | 42 | 48 | 10 | 48 | 0.81 | 0.50 | 0.68 | single-center | Yes | DL | contrast-enhanced | pCR | Yes | No | No |  | 3D |
| Ye(ex)(pre-dl) | 2025 | China | 30 | 34 | 22 | 62 | 0.58 | 0.65 | 0.63 | single-center | Yes | DL | contrast-enhanced | pCR | Yes | No | No |  | 3D |
| Ye(ex)(pre-hi) | 2025 | China | 39 | 45 | 13 | 51 | 0.75 | 0.53 | 0.67 | single-center | Yes | DL | contrast-enhanced | pCR | Yes | No | No |  | 3D |
| Ye(ex)(post-rad) | 2025 | China | 40 | 33 | 12 | 63 | 0.77 | 0.66 | 0.71 | single-center | Yes | DL | contrast-enhanced | pCR | Yes | No | No |  | 3D |
| Ye(ex)(post-dl) | 2025 | China | 25 | 32 | 27 | 64 | 0.48 | 0.66 | 0.57 | single-center | Yes | DL | contrast-enhanced | pCR | Yes | No | No |  | 3D |
| Ye(ex)(post-hi) | 2025 | China | 36 | 29 | 16 | 67 | 0.69 | 0.70 | 0.72 | single-center | Yes | DL | contrast-enhanced | pCR | Yes | No | No |  | 3D |
| Ye(ex)(delta-rad) | 2025 | China | 39 | 32 | 13 | 64 | 0.75 | 0.67 | 0.73 | single-center | Yes | DL | contrast-enhanced | pCR | Yes | No | No |  | 3D |
| Ye(ex)(delta-dl) | 2025 | China | 34 | 38 | 18 | 58 | 0.65 | 0.60 | 0.60 | single-center | Yes | DL | contrast-enhanced | pCR | Yes | No | No |  | 3D |
| Ye(ex)(delta-hi) | 2025 | China | 39 | 38 | 13 | 58 | 0.75 | 0.60 | 0.70 | single-center | Yes | DL | contrast-enhanced | pCR | Yes | No | No |  | 3D |
| Ye(ex)(nicerα) | 2025 | China | 35 | 25 | 17 | 71 | 0.67 | 0.74 | 0.73 | single-center | Yes | DL | contrast-enhanced | pCR | Yes | No | No |  | 3D |
| Ye(ex)(post) | 2025 | China | 35 | 17 | 17 | 79 | 0.67 | 0.82 | 0.78 | single-center | Yes | DL | contrast-enhanced | pCR | Yes | No | No |  | 3D |
| Ye(ex)(delta) | 2025 | China | 36 | 20 | 16 | 76 | 0.69 | 0.79 | 0.77 | single-center | Yes | DL | contrast-enhanced | pCR | Yes | No | No |  | 3D |
| Ye(ex)(nicerδ) | 2025 | China | 39 | 12 | 13 | 84 | 0.75 | 0.87 | 0.87 | single-center | Yes | DL | contrast-enhanced | pCR | Yes | No | No |  | 3D |
| Zheng(ex)(pre-treat) | 2025 | China | 16 | 10 | 14 | 19 | 0.54 | 0.67 | 0.65 | multi-center | Yes | DL | CT | MPR | Yes | No | No | 22 | 3D |
| Zheng(ex)(pre-surgery) | 2025 | China | 17 | 8 | 13 | 21 | 0.58 | 0.71 | 0.69 | multi-center | Yes | DL | CT | MPR | Yes | No | No | 22 | 3D |
| Zheng(ex)(Mobile-dual) | 2025 | China | 17 | 8 | 13 | 21 | 0.58 | 0.73 | 0.77 | multi-center | Yes | DL | CT | MPR | Yes | No | No | 22 | 3D |
| Zheng(ex)(ResNet-dual) | 2025 | China | 21 | 8 | 9 | 21 | 0.70 | 0.73 | 0.70 | multi-center | Yes | DL | CT | MPR | Yes | No | No | 22 | 3D |
| Zheng(ex)(One-dual) | 2025 | China | 18 | 9 | 12 | 20 | 0.62 | 0.70 | 0.69 | multi-center | Yes | DL | CT | MPR | Yes | No | No | 22 | 3D |
| Zheng(ex)(dual+c) | 2025 | China | 19 | 3 | 11 | 26 | 0.63 | 0.90 | 0.79 | multi-center | Yes | DL | CT | MPR | Yes | Yes | No | 22 | 3D |
| Zheng(ex)(dual+rad) | 2025 | China | 18 | 8 | 12 | 21 | 0.59 | 0.73 | 0.78 | multi-center | Yes | DL | CT | MPR | Yes | No | No | 22 | 3D |

Note: TP, ture positive, TN, ture negative, ML, machine learning, FN, false negative, FP, false positive, DL, deep learning.
